# Low-Temperature Oxidation Induced Phase Evolution with Gradient Magnetic Heterointerfaces for Superior Electromagnetic Wave Absorption

**DOI:** 10.1007/s40820-024-01516-z

**Published:** 2024-09-22

**Authors:** Zizhuang He, Lingzi Shi, Ran Sun, Lianfei Ding, Mukun He, Jiaming Li, Hua Guo, Tiande Gao, Panbo Liu

**Affiliations:** 1 School of Chemistry and Chemical Engineering, Northwestern Polytechnical University, Xi’an, 710129 People’s Republic of China; 2 School of Marine Science and Technology, Northwestern Polytechnical University, Xi’an, 710072 People’s Republic of China

**Keywords:** Magnetic heterointerfaces, Phase evolution, Interfacial polarization, Magnetic coupling, Electromagnetic wave absorption

## Abstract

Gradient magnetic heterointerfaces have injected infinite vitality in optimizing impedance matching, adjusting dielectric/magnetic resonance and promoting electromagnetic (EM) wave absorption, but still exist a significant challenging in regulating local phase evolution. Herein, accordion-shaped Co/Co_3_O_4_@N-doped carbon nanosheets (Co/Co_3_O_4_@NC) with gradient magnetic heterointerfaces have been fabricated via the cooperative high-temperature carbonization and low-temperature oxidation process. The results indicate that the surface epitaxial growth of crystal Co_3_O_4_ domains on local Co nanoparticles realizes the adjustment of magnetic-heteroatomic components, which are beneficial for optimizing impedance matching and interfacial polarization. Moreover, gradient magnetic heterointerfaces simultaneously realize magnetic coupling, and long-range magnetic diffraction. Specifically, the synthesized Co/Co_3_O_4_@NC absorbents display the strong electromagnetic wave attenuation capability of − 53.5 dB at a thickness of 3.0 mm with an effective absorption bandwidth of 5.36 GHz, both are superior to those of single magnetic domains embedded in carbon matrix. This design concept provides us an inspiration in optimizing interfacial polarization, regulating magnetic coupling and promoting electromagnetic wave absorption.

**Supplementary Information:**

The online version contains supplementary material available at 10.1007/s40820-024-01516-z.

## Highlights

- Co/Co_3_O_4_@NC nanosheets with gradient magnetic heterointerfaces have been fabricated by the high-temperature carbonization/low-temperature oxidation processes.
- Experimental and theoretical simulation results indicate that magnetic heterointerfaces engineering is beneficial for optimizing impedance matching and promoting electromagnetic wave absorption.
- Gradient magnetic heterointerfaces with magnetic-heteroatomic components realize the adjustment of interfacial polarization, magnetic coupling, and long-range magnetic diffraction.

**Supplementary Information:**

The online version contains supplementary material available at 10.1007/s40820-024-01516-z.

## Graphical abstract

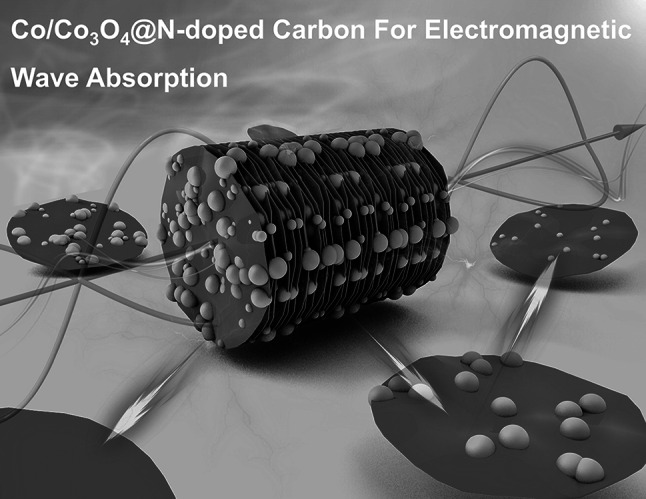

## Introduction

The popularization of wireless communication technology, especially the explosive growth and implementation of 5G technology, greatly promotes the upgrading of global industries and the development of the economy and society [1–3]. However, technological advancements often bring greater challenges. The emergence of electromagnetic (EM) radiation and the responding pollution inevitably affects the normal operation of electronic devices and the health of human beings [4–6]. Therefore, fabricating efficient EM wave absorbents with thin, light, strong absorption and wide broadband has become the promising and effective solution to address these issues [7–11]. Based on these requirements, many strategies have been proposed to construct high-performance EM wave absorbents [12–14]. The magnetic–dielectric synergistic effect is a classic theory which is usually used to elucidate the mechanism of EM wave attenuation [15]. Based on the theoretical research, the focus has gradually shifted to using magnetic and nonmagnetic components to regulate and improve the absorption intensity and effective absorption bandwidth [16–19]. However, among these methods, hydrothermal treatment and etching are usually required, which greatly limit the mass production of materials and introduce more uncertainty.

In recent years, metal–organic frameworks (MOFs) and their derivatives have been considered as the most promising candidates in EM wave absorption due to their tunable chemical composition, mesoporous properties, and diverse microstructures [20–24]. As early as 2015, Du et al. firstly used Prussian blue as a precursor to synthesize Fe/C nanocubes through a one-step pyrolysis method, which opened a new era in the field of EM wave absorption for MOFs derivatives [25]. After that, various morphologies of MOFs derivatives have been employed as EM wave absorbents [26–31]. However, few researchers focus on constructing MOFs derivatives via the manipulation of pyrolysis process, and the mechanism of structural design and EM wave absorption performance has not been clarified. Besides, due to the larger magnetic force between single magnetic nanoparticles, they are preferred to agglomerate to form larger magnetic domains during the pyrolysis process. To address this issue, constructing hollow nanoparticles or yolk–shell structures with coexisting micro- and mesopores has been proposed to reduce material density and improve skin depth. It is well known that the prominence of EM wave absorbing materials prepared through the direct pyrolysis of single MOFs has declined due to the inherent limitations of non-tunability and the singularity of a single-component system. To address these challenges, there is an imperative need to develop MOF-derived carbon materials that integrate structural design and component control, thereby streamlining the preparation process.

Herein, Co/Co_3_O_4_@NC nanosheets with gradient magnetic heterointerfaces have been fabricated by the high-temperature carbonization/low-temperature oxidation processes. Experimental data and simulation results indicate that the generation of gradient magnetic heterointerfaces is beneficial for optimizing impedance matching and EM wave absorption, realizing the adjustment of interfacial polarization, magnetic coupling and long-range magnetic diffraction. As expected, when the filler ratio is 25 wt%, the optimal reflection loss is − 53.5 dB and the bandwidth reaches 5.36 GHz. This study is the pioneer to investigate the internal relationship between gradient magnetic heterointerfaces and EM wave absorption attenuation, which provided a new theoretical basis to pursue high-efficiency EM wave absorbents by magnetic heterointerfaces engineering.

## Experimental Section

### Synthesis of Accordion-Shaped ZIF Precursors

In a typical synthesis, 6 mmol of dimethylimidazole and 0.5 mmol of Co(OAc)_2_·4H_2_O were dissolved in 20 mL of deionized water and stirred for 24 h. The resulting accordion-shaped ZIF precursors were collected by centrifugation, washed with ethanol several times, and dried in a vacuum oven at 60 °C for 24 h.

### Synthesis of Co@N-Doped Carbon (Co@NC) Nanosheets

The obtained accordion-shaped ZIF precursors were calcined at 800 °C for 3 h under Ar atmosphere, resulting in the formation of Co@NC nanosheets.

### Synthesis of Co/Co_3_O_4_@N-Doped Carbon (Co/Co_3_O_4_@NC) Nanosheets

The obtained Co@NC nanosheets were calcined at 230 °C for 3 h under Air atmosphere, yielding to the formation of Co/Co_3_O_4_@NC nanosheets.

### Synthesis of Co_3_O_4_@N-Doped Carbon (Co_3_O_4_@NC) Nanosheets

The obtained accordion-shaped ZIF precursors were calcined at 320 °C for 3 h under Air atmosphere, resulting in the formation of Co_3_O_4_@NC nanosheets.

### Characterizations

The microstructures were imaged by scanning electron microscopy (SEM, FEI Verios G4). The high-resolution morphologies and elemental mapping section were obtained by transmission electron microscopy (TEM, FEI Talos F200X). X-ray diffraction (XRD) data containing the information of crystal structures were characterized by a Bruker-D8-DISCOVER X-ray diffractometer. The surface chemical composition and valence state of elements were obtained by a Phoibos-100-spectrometer X-ray photoelectron spectrometer (XPS). The static magnetic properties were characterized by vibrating sample magnetometer (VSM, LakeShore7404). The reflection loss (*R*_L_) values, impedance match degree (Z_in_/Z_0_), radar cross section (RCS) simulation, and computational analysis were presented in the Supporting Information.

## Results and Discussions

**Fig. 1:**
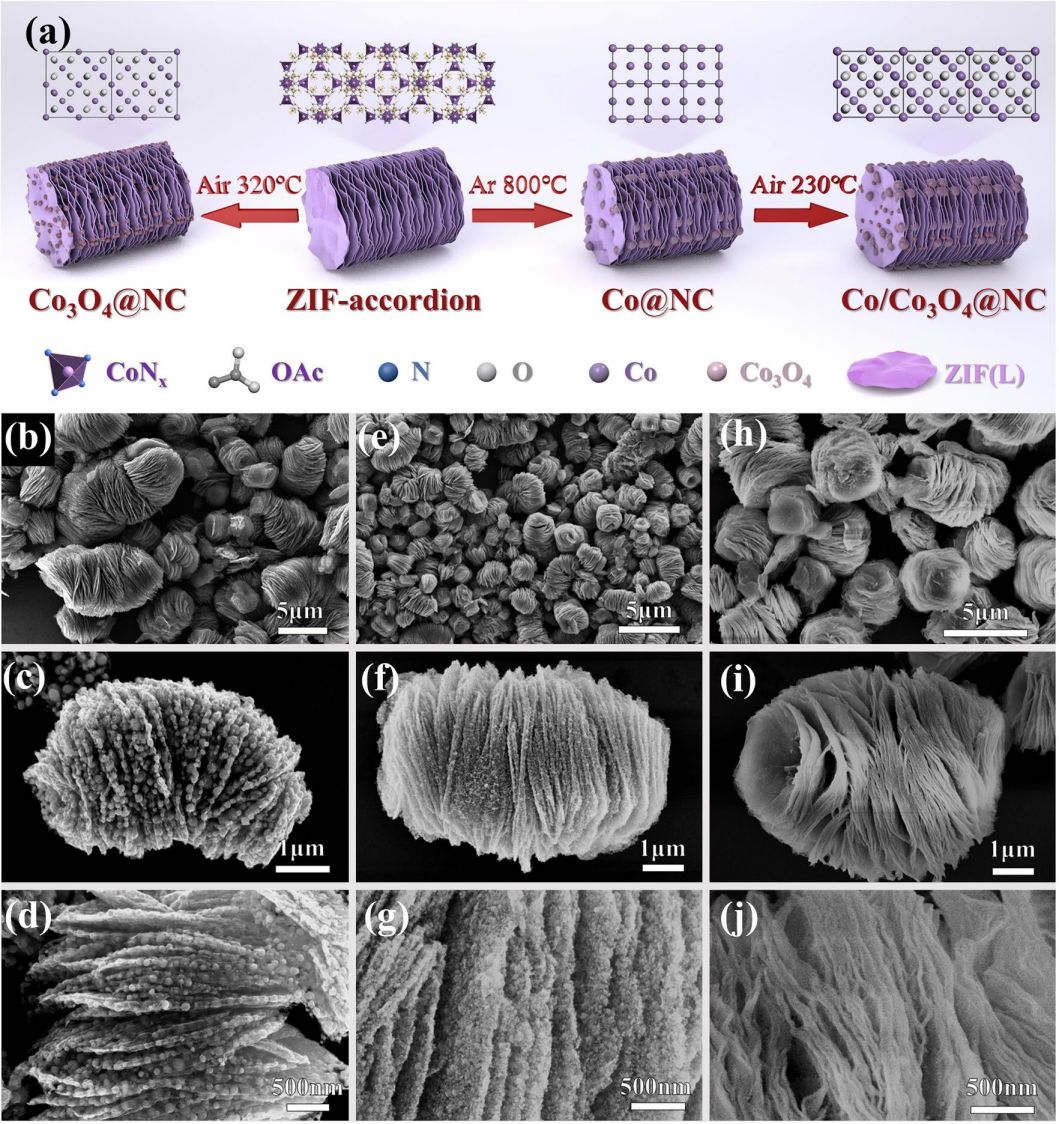
**a** Schematic illustration of the synthetic processes, SEM images of **b–d** Co@NC, **e–g** Co/Co_3_O_4_@NC, **h–j** Co_3_O_4_@NC

The synthesized processes of Co@NC, Co/Co_3_O_4_@NC, and Co_3_O_4_@NC nanosheets are illustrated in Fig. 1a. First, accordion-shaped ZIF precursors with multilayer nanosheets were synthesized by the co-precipitation method (Fig. S1a) [32]. TEM and the corresponding element mapping images indicate that C, N, O, and Co elements are uniformly distributed in the accordion-shaped ZIF. As shown in Fig. S2, the diffraction peaks of the obtained ZIF precursors are consistent with the simulation results. Subsequently, the obtained precursors were annealed in a tubular furnace under different annealing temperatures and atmospheres, resulting in the phase evolution with gradient magnetic heterointerfaces [33–35]. Under high-temperature argon and low-temperature air environment, Co@NC nanosheets with Co phase (Fig. 1b–d) and Co_3_O_4_@NC nanosheets with Co_3_O_4_ semiconductor phase (Fig. 1h–j) are generated, respectively. For Co@NC nanosheets, these reduced Co domains are preferred to agglomerate to form larger magnetic nanoparticles due to the larger magnetic force [36], thus the average size of Co nanoparticles is in the range of 120–180 nm (Fig. S3). For Co_3_O_4_@NC nanosheets, it is clear that small Co_3_O_4_ nanoparticles are embedded in carbon nanosheets, and the average size of Co_3_O_4_ nanoparticles is only about 8 nm (Fig. S4). By the cooperative high-temperature carbonization and low-temperature oxidation, the surface epitaxial growth of crystal Co_3_O_4_ phase on local Co phase is realized (Fig. 1e–g), and the phase evolution inevitably decreases the size of Co nanoparticles, as shown in Fig. S5.

**Fig. 2:**
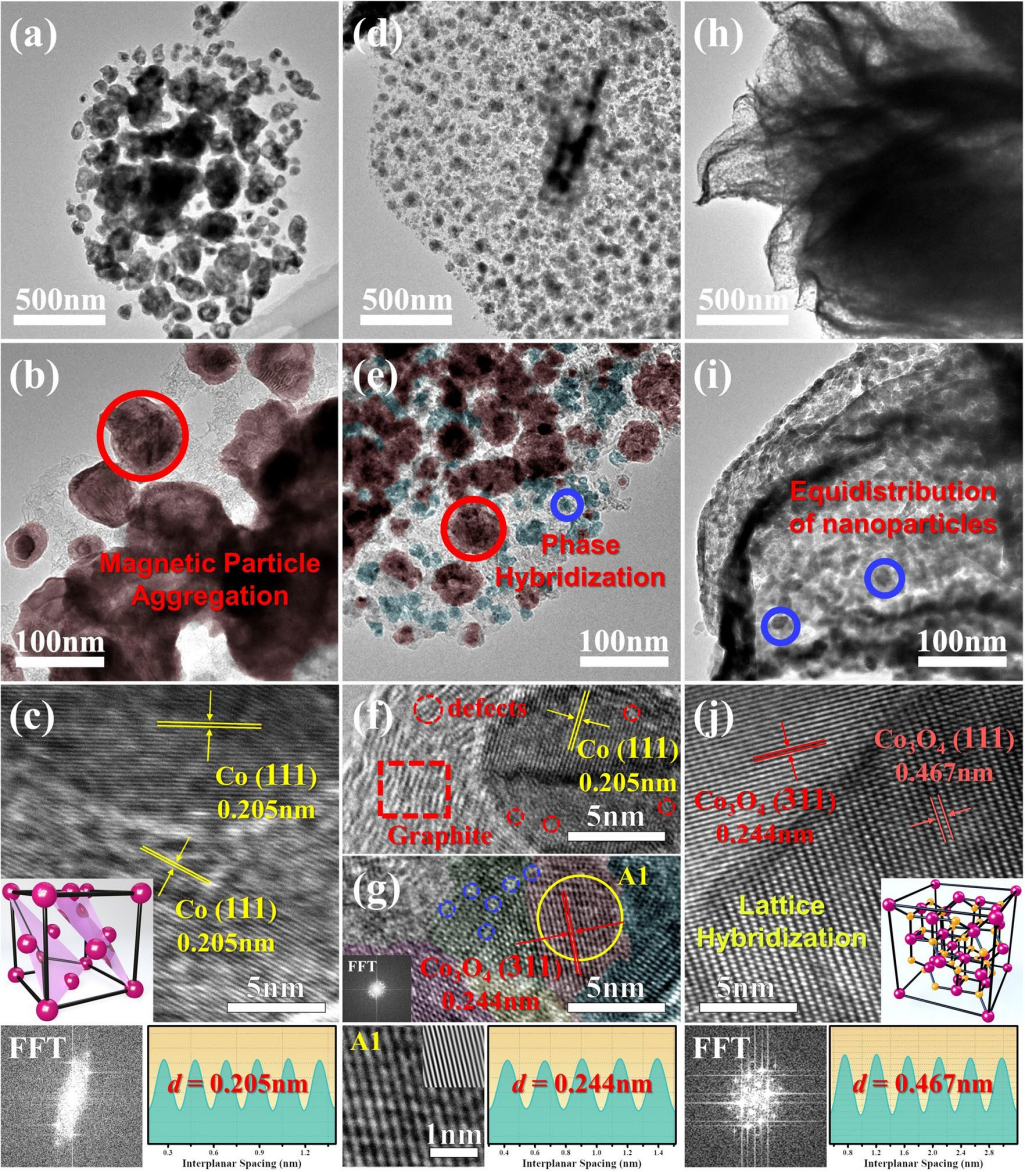
TEM and HRTEM images of **a–c** Co@NC, **d–g** Co/Co_3_O_4_@NC, **h–j** Co_3_O_4_@NC

TEM and HRTEM images of Co@NC, Co/Co_3_O_4_@NC, and Co_3_O_4_@NC nanosheets are shown in Fig. 2. Obviously, Fig. 2a, b further confirms the phenomenon of Co nanoparticles in Co@NC, while small Co_3_O_4_ nanoparticles are uniformly distributed on the carbon nanosheets for Co_3_O_4_@NC (Fig. 2h, i), and the size of Co nanoparticles decreases due to the presence of Co_3_O_4_ for Co/Co_3_O_4_@NC (Fig. 2d, e). In Fig. 2c, HRTEM image shows that the lattice of 0.205 nm corresponds to the (111) plane of Co. Based on the polarization resonance theory, different planes or orientations usually lead to modified electronic bands and intracrystalline interface coupling, thus inducing discrepant band realignment to enhance dipole relaxation and interfacial polarization. Figure 2f illustrates that these reduced Co nanoparticles are completely wrapped by the graphite carbon layer under high-temperature conditions, exposing a large number of defects (Fig. S6). The pseudo-color image in Fig. 2g provides a more intuitive explanation of the presence of crystal hybridization in Co_3_O_4_ nanoparticles. The presence of multi-oriented crystal planes can generate a large number of point defects, thereby enhancing dipole polarization and interfacial polarization. Clear Moiré fringes can also be observed in Fig. 2j, which are caused by the (311) and (111) crystal planes of Co_3_O_4_.

**Fig. 3:**
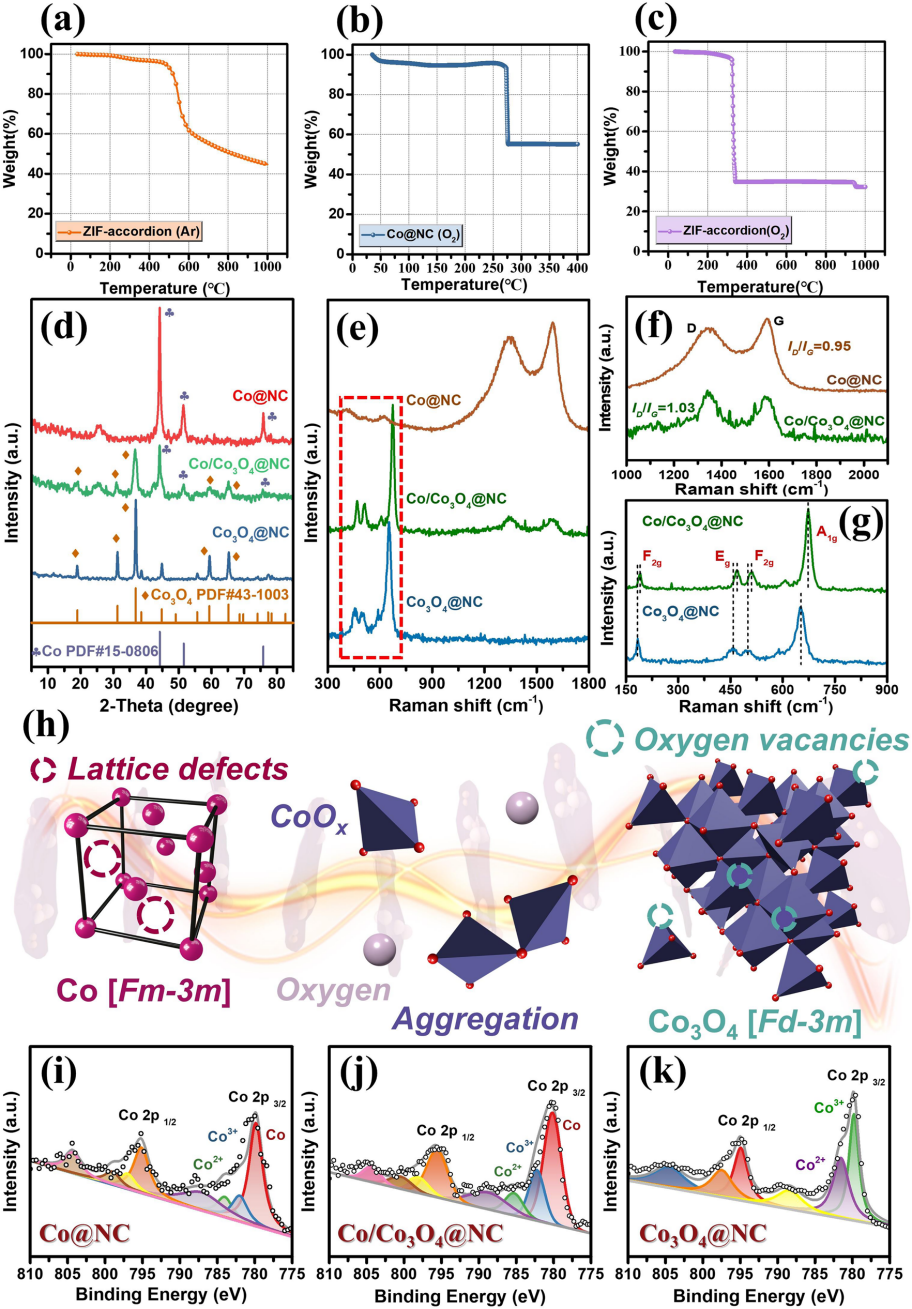
**a–c** Thermogravimetric curve, **d** XRD pattern, **e** Raman full spectrum,** f** amplified D and G peaks, **g** Raman peaks of spinel Co_3_O_4_, **h** phase transition, XPS pattern of Co 2*p* of **i** Co@NC, **j** Co/Co_3_O_4_@NC, **k** Co_3_O_4_@NC

Figure 3a–c shows the thermogravimetric curves of the ZIF precursors under argon and air conditions, Co@NC under air conditions, respectively. At low temperature, the mass of all samples shows a slow downward trend, caused by the evaporation of water vapor adsorbed on the surface and the thermal decomposition of surface groups. As expected, around 450 °C, ZIF precursors begin to decompose on a large scale, and the pyrolysis rate reaches a constant state at 610 °C. The obtained Co@NC is pyrolyzed at 800 °C, and testing is conducted within the temperature range of 30–400 °C. As the temperature increasing to around 270 °C, the amorphous/graphite carbon derived from the material begins to oxidize and evaporate as CO_2_, resulting in a sharp decrease in sample mass. At around 320 °C, the ZIF precursors begins to oxidize, and the organic framework in the material is oxidized and destroyed, promoting the complete conversion from Co^2+^ to Co_3_O_4_. XRD patterns of the obtained Co@NC, Co/Co_3_O_4_@NC, and Co_3_O_4_@NC are presented in Fig. 3d. Among them, the three strong diffraction peaks at 44.2°, 51.5°, and 75.9° correspond to the (111), (200), and (220) crystal planes of Co (PDF#15–0806) [37], respectively. The three diffraction peaks of 19.0°, 31.2°, and 36.8° correspond to the (111), (220), and (311) crystal planes of Co_3_O_4_ (PDF#43–1003) [38], respectively. Surprisingly, the diffraction peaks of the products match well with the standard card and corresponded to lattice fringes of different sizes in HRTEM. Specifically, due to the catalytic effect of magnetic Co particles, the diffraction peak at 26.3° is attributed to the formation of graphite [39, 40]. Due to the low temperature, the degree of graphitization cannot observe in Co_3_O_4_@NC, resulting in the disappearance of the 26.3° diffraction peak. Figure 3e shows the degree of graphitization of the carbon skeleton using Raman spectroscopy. Compared with Co@NC and Co/Co_3_O_4_@NC, the disappearance of D and G peaks in Co_3_O_4_@NC further confirms the absence of graphite carbon and amorphous carbon. The *I*_D_/*I*_G_ value of Co@NC is 0.95, indicating that the graphite carbon catalyzed by the pure Co phase leads to a higher degree of graphitization (Fig. 3f). In addition, C atoms undergo structural damage during pyrolysis under air conditions, thus the degree of defects in Co/Co_3_O_4_@NC increases, leading to an increase of *I*_D_*/I*_G_ value. The magnified Raman peak of Co_3_O_4_ is shown in Fig. 3g. Clearly, the peaks at 184, 458, 503, and 652 cm^−1^ correspond to the F_2g_, E_g_, F_2g_, and A_1g_ vibration modes of Co_3_O_4_, respectively. The F_2g_ vibration mode has the lowest symmetry and involves more complex atomic vibrations in the crystal. A_1g_ is a highly symmetric vibration mode, while E_g_ involves the relative motion of adjacent atoms in the crystal. These three classic vibration modes demonstrate the precise synthesis of Co_3_O_4_. Compared with Co_3_O_4_@NC, owing to the epitaxial growth of Co_3_O_4_ along the surface of Co particles, the classical spinel structure is doped. As shown in Fig. 3g, the shift of the vibration peak proves this point [5, 41–44]. As shown in Fig. S7, the XPS spectra display strong signals of C 1*s*, N 1*s*, O 1*s*, and Co 2*p* elements, which are highly consistent with the previous characterization results. Notably, the signal of N 1*s* in Co_3_O_4_@NC was not displayed, which is attributed to the disappearance of a large amount of the carbon layer due to the oxidation (Fig. S8). Figure 3i–k shows the fine spectra of Co in the Co@NC, Co/Co_3_O_4_@NC, and Co_3_O_4_@NC. Co 2*p* can be split into Co 2*p*_3/2_ and Co 2*p*_1/2_. The shift of characteristic peaks is attributed to the different ways in which Co elements exist within their systems. As expected, Co@NC only contains a portion of Co^3+^ (782.1 eV) and Co^2+^ (784.2 eV), which is attributed to the oxidation of Co elemental exposed to air. Compared with Co@NC, the Co^3+^ (782.4 eV) and Co^2+^ (785.2 eV) content of Co/Co_3_O_4_@NC significantly increased, indicating the successful growth of Co_3_O_4_. There is no peak of Co elemental in Co_3_O_4_@NC, which only display the peaks of Co^3+^ (780.2 eV) and Co^2+^ (782.6 eV), perfectly corresponding to the different valence states of Co ions in the surface and inner layers of the spinel structure, indicating that a pure Co_3_O_4_ phase is obtained.

**Fig. 4:**
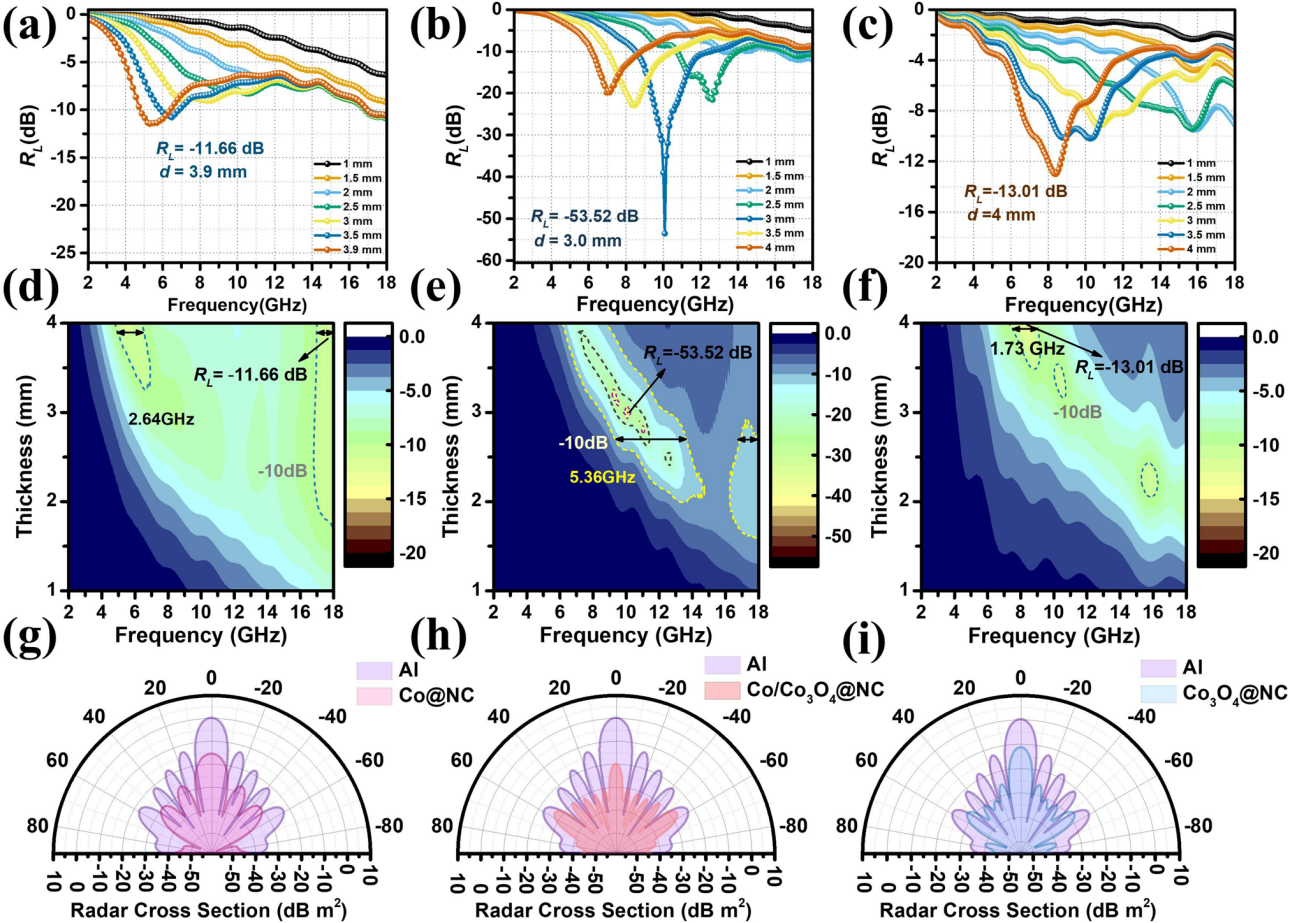
*R*_L_ values, 2D colormap and radar cross section of **a, d, g** Co@NC, **b, e, h** Co/Co_3_O_4_@NC and **c, f, i** Co_3_O_4_@NC

The reflection loss (*R*_*L*_) of Co@NC, Co/Co_3_O_4_@NC, Co_3_O_4_@NC is characterized by transmission line theory and the results with a loading ratio of 25 wt% are shown in Fig. 4. Obviously, Co@NC (Fig. 4a, d) and Co_3_O_4_@NC (Fig. 4c, f) exhibits poor *R*_*L*_ values and unsatisfied effective absorption bandwidth. As expected, multiphase hybridization engineering effectively provides plenty of heterogeneous interfaces and defects, leading to significant EM wave attenuation. Specifically, the minimum *R*_*L*_ value of Co/Co_3_O_4_@NC (Fig. 4b, e) is up to − 53.5 dB at 3.0 mm, and the effective absorption bandwidth below − 10 dB reaches 5.36 GHz. This conclusion indicates that designing multiphase structures is an effective strategy for improving EM wave absorption [45]. The mechanism of enhancing EM wave absorption performance can be explained by impedance matching [46]. Generally speaking, the area of |Z_in_/Z_0_| should be close to 1, representing the best impedance matching. It can be observed that the stripe area of Co/Co_3_O_4_@NC is larger than that of Co@NC and Co_3_O_4_@NC (Fig. S9), representing a good matching of impedance characteristics. Furthermore, the impedance area of the Co/Co_3_O_4_@NC stripe close to 1 overlap with the area of *R*_*L*_ ≤ − 10 dB. This not only means that impedance matching is optimal but also indicates that Co/Co_3_O_4_@NC has the strongest EM wave absorption performance. To assist in proving impedance matching, Z_in_ is decomposed according to Z_in_ = Z'- jZ'' to obtain Z' and Z''. In theory, when Z' = 1 and Z'' = 0, it represents the best impedance matching. From Fig. S10, it can be seen that Co/Co_3_O_4_@NC at the frequency corresponds to its minimum *R*_*L*_ value point, the above theory is satisfied. At a filler ratio of 25 wt%, the impedance matching of Co/Co_3_O_4_@NC is the best, and the EM wave absorption performance is the strongest [47].

To further demonstrate the performance of EM wave absorbing materials in the real far-field domain, pure aluminum plates (PEC layers) are simulated using HFSS [48–50]. Here, an aluminum plate with a size of 180 × 180 × 5 mm^3^ is used as the substrate, and three materials with a mass ratio of 25 wt% are coated on the surface of the PEC layer. The external layer is set to X and Y is a perfect matching layer of 200 mm. The composite materials of Co@NC, Co/Co_3_O_4_@NC, and Co_3_O_4_@NC are set to 28.9, 28.0, and 29.0 mm, respectively. As shown in Fig. 4g–i, the simulation result of radar cross section (RCS) is Co/Co_3_O_4_@NC < Co@NC < Co_3_O_4_@NC, which is consistent with the test results. In addition, compared with the RCS values of aluminum plates within the range of − 90° < θ < 90°, the RCS values of three composite materials are significantly reduced, compared with pure aluminum plates. Specifically, Co/Co_3_O_4_@NC and the Al plate reached 19.8 dB m^2^ at 0°. In the final analysis, the low *R*_L_ value, high matching degree, and low RCS value exhibited by Co/Co_3_O_4_@NC composite materials mean they can act as the promising candidates for EM wave absorbing materials.

**Fig. 5:**
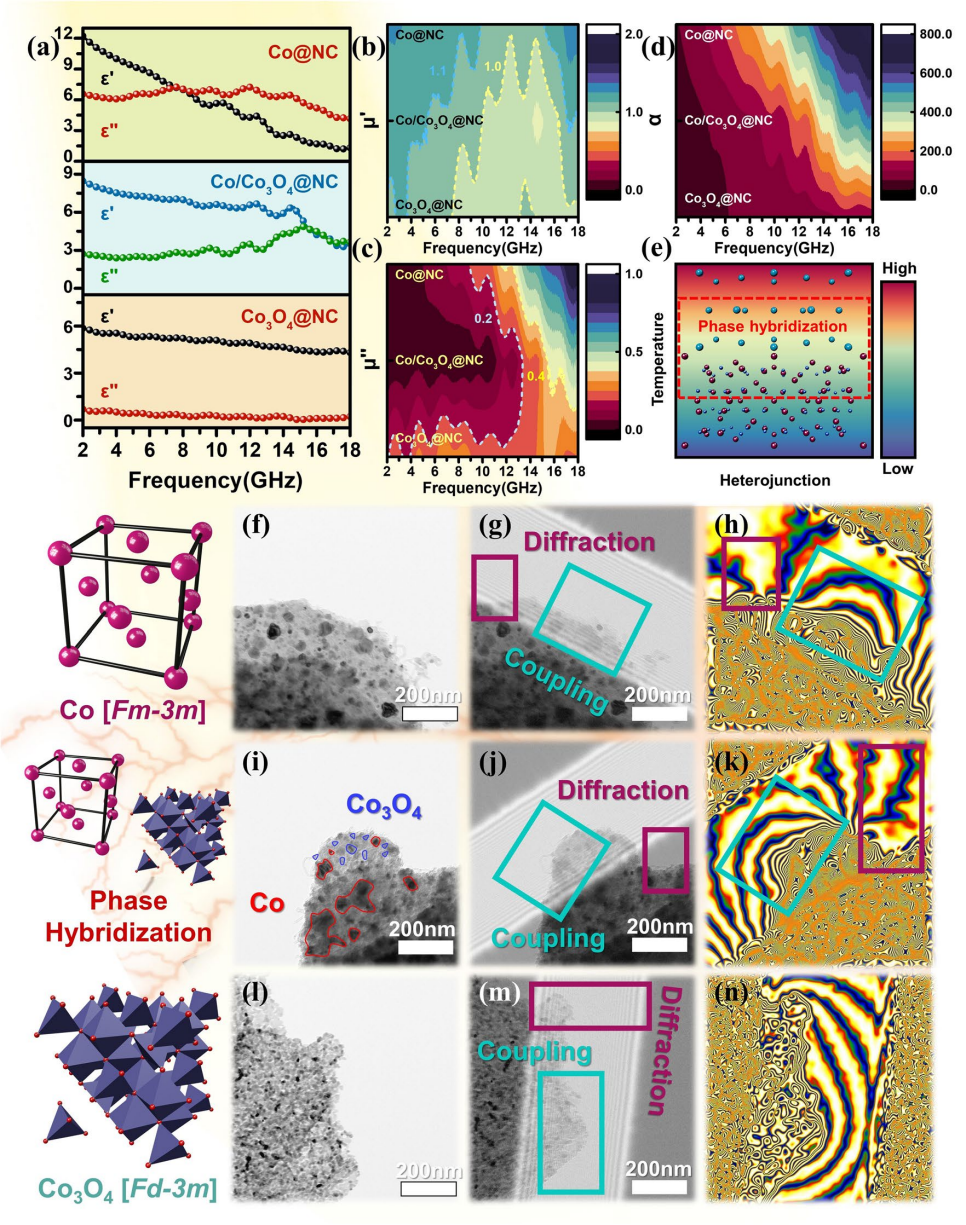
**a–c** Electromagnetic parameters, **d** attenuation constant, TEM, electronic holography, and magnetic coupling diagrams of **f, i, l** Co@NC, **g, j, m** Co/Co_3_O_4_@NC and **h, k, n** Co_3_O_4_@NC, the phase hybridization diagram of **e** Co/Co_3_O_4_@NC

It is well known that the EM wave absorption performance depends on the complex permittivity and complex permeability. The *ε’* and *ε”* values represent the storage and loss of dielectric energy, respectively. The overall dielectric loss capacity is determined by the dielectric loss tangent (*tanδ*_*ε*_ = *ε”*/*ε’*). Figure 5a shows the *ε’* and *ε”* values of Co@NC, Co/Co_3_O_4_@NC and Co_3_O_4_@NC. Obviously, the *ε’* values of all samples decrease with increase in the frequency. Owing to the increase in high-frequency polarization hysteresis, multiple resonance peaks appear in the range of 2–18 GHz for *ε”*, indicating the generation of a frequency dispersion effect. Figure S10 shows the dielectric loss tangent values. It is clear that the *tanδ*_*ε*_ values of Co@NC and Co/Co_3_O_4_@NC are all higher than that of Co_3_O_4_@NC. This phenomenon can be explained by the higher conductivity of graphite compared to Co_3_O_4_ semiconductor phase [51, 52].

Magnetic loss is identified as a critical factor in determining the EM wave absorption performance. Figure 5b, c shows the *μ’* and *μ’’* values of Co@NC, Co/Co_3_O_4_@NC, and Co_3_O_4_@NC. It is widely acknowledged that the variation of *μ’’* is closely related to the composition of magnetic nanoparticles. Nevertheless, *μ’* is less affected by its composition. Apparently, the *μ’’* value of Co@NC is higher than Co/Co_3_O_4_@NC and Co_3_O_4_@NC, which is attributed to the aggregation of large-sized Co particles, resulting in strong magnetic loss. The magnetic properties are analyzed using vibrating sample magnetometer (VSM), as shown in Fig. S11. Owing to the presence of semiconductors, the saturation magnetization intensity (*M*_*S*_) values of Co/Co_3_O_4_@NC and Co_3_O_4_@NC are only 18.92 and 0.61 emu g^−1^, which are lower than Co@NC (50.95 emu g^−1^). The magnetic coercivity (*H*_*C*_) values of Co@NC, Co/Co_3_O_4_@NC, and Co_3_O_4_@NC are 371.26, 541.33, and 41.62 Oe, respectively. The decrease in hysteresis loss (Fig. S12) is attributed to the introduction of the semiconductor Co_3_O_4_, which also confirm that Co@NC and Co_3_O_4_@NC possess the maximum and minimum magnetic loss tangent angles, respectively. For Co@NC and Co/Co_3_O_4_@NC, considering eddy current losses is another critical factor in determining magnetic losses. As depicted in Fig. S13, the *μ’’*(*μ’*)^−2^*f* ^−1^ values for both Co@NC and Co/Co_3_O_4_@NC fluctuate within the range of 2–18 GHz, indicating that ferromagnetic resonance and eddy current loss simultaneously contribute to magnetic loss. Figure 5e shows the relationship between temperature and spinel semiconductors, the incorporation of the spinel semiconductor has led to a decrease in both electrical conductivity and magnetic permeability, while also eliminating high-frequency eddy currents, resulting in a pronounced absorption peak at high frequencies for Co/Co_3_O_4_@NC. Consequently, this elucidates why Co@NC exhibits the highest attenuation constant (*α*), thus its absorption performance is inferior to that of Co/Co_3_O_4_@NC, which has a lower *α* value (Fig. 5d).

**Fig. 6:**
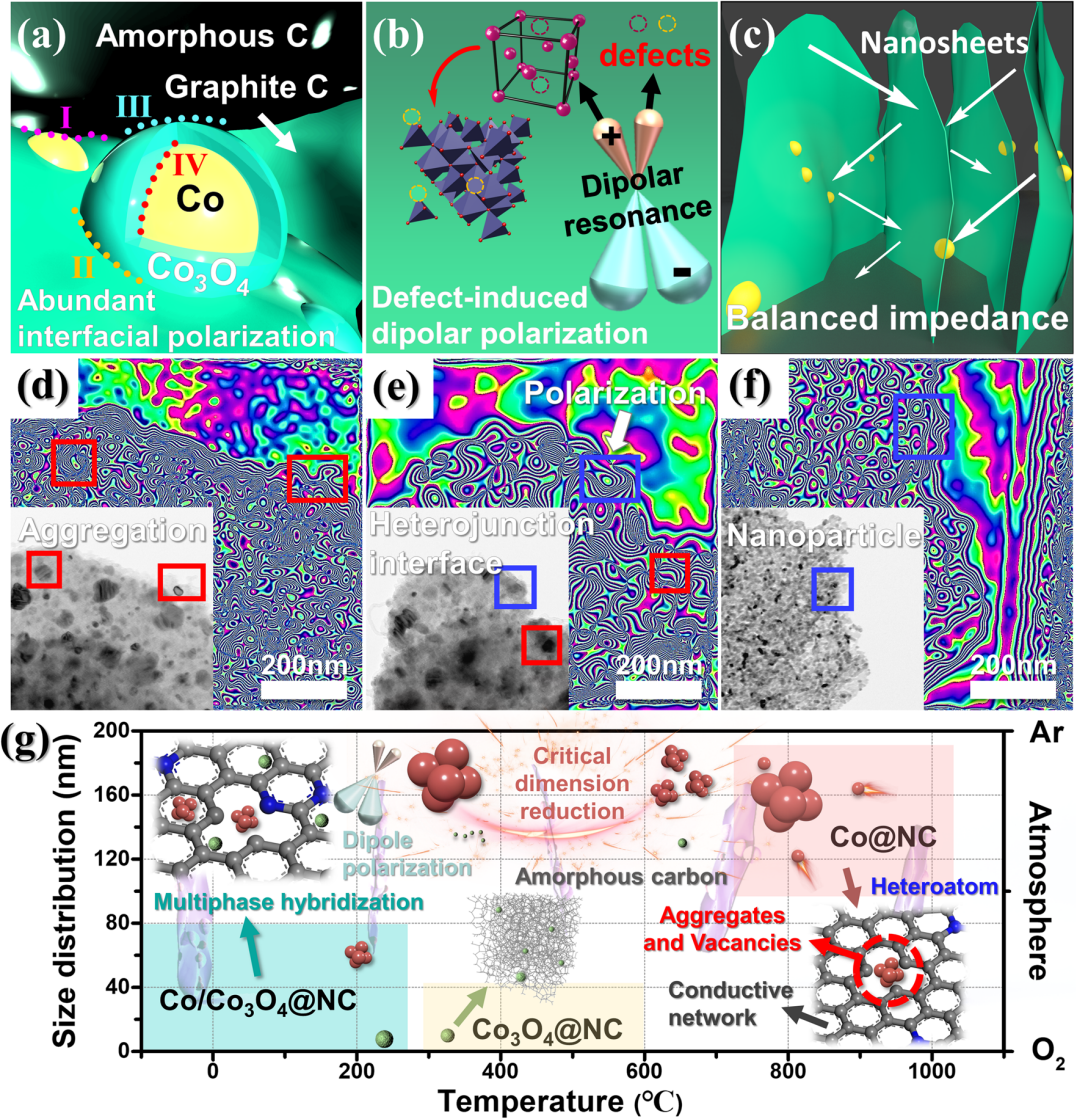
**a–c** Structural modes and **d–f** holograms of Co@NC, Co/Co_3_O_4_@NC and Co_3_O_4_@NC,** g** the possible electromagnetic wave absorption mechanism

The results indicate that the EM wave absorption performance can be modulated by the phase evolution and magnetic heterointerfaces engineering. Magnetic nanoparticles exceeding the critical size not only generate magnetic resonance and magnetic coupling, but also cause long-range magnetic diffraction in adjacent magnetic domains [53]. As shown in Fig. 5f–n, the stray magnetic flux lines indicate that magnetic loss is related to the aggregation of Co nanoparticles. Each microscale magnetic domain represents a sole domain and acts as high-density magnetic activation antennas to radiate out stray diffraction flux lines to interact with other magnetic domains by means of magnetic coupling and long-range magnetic diffraction. Therefore, magnetic coupling and long-range magnetic diffraction simultaneously establish a connected magnetic network to interfere with incident EM wave, dissipating the loss of incidence EM wave. However, the introduction of Co_3_O_4_ nanoparticles could weak the aggregation of Co nanoparticles, thus the decreased size of Co nanoparticles leads to short-range magnetic exchange interactions. The coexistence of multiple loss mechanism also contributes to the improvement of EM wave absorption performance. Figure 5n clearly shows that there are fewer magnetic flux lines in Co_3_O_4_@NC. Due to the weak magnetism of Co_3_O_4_, its loss mode is weaker than that of the other absorbents. The possible loss structural modes of Co@NC, Co/Co_3_O_4_@NC, and Co_3_O_4_@NC are proposed in Fig. 6a–c. It is distinct that multiple polarization loss and matched impedance provided by the nanosheets are key to the final EM wave performance [54, 55]. To support this conclusion, the holograms of Co@NC, Co/Co_3_O_4_@NC, and Co_3_O_4_@NC are shown in Fig. 6d–f. The rich variety of crystal phases and abundant heterogeneous interfaces, defects, and vacancies suggest that Co/Co_3_O_4_@NC provides excellent EM wave absorption performance compared with that of Co@NC and Co_3_O_4_@NC (Fig. 6g). The simplification of the preparation method makes mass production possible, providing an excellent candidate for future industrial applications of EM wave absorption materials.

## Conclusion

In conclusion, accordion-shaped Co/Co_3_O_4_@NC nanosheets with gradient magnetic heterointerfaces have been successfully synthesized via the cooperative high-temperature carbonization and low-temperature oxidation process. The results indicate that the generation of Co_3_O_4_ domains on local Co nanoparticles can adjust the magnetic-heteroatomic components, which are beneficial for the enhancement of interfacial polarization and EM synergy. Ultimately, the Co/Co_3_O_4_@NC nanosheets achieve a minimum reflection loss value of − 53.5 dB and an effective absorption bandwidth of 5.36 GHz. This simple preparation method provides a valuable insight for the efficient mass production of absorbing agents and stimulates us an inspiration in adjusting EM wave absorption.

## Supplementary Information

Below is the link to the electronic supplementary material.

Supplementary file1 (DOCX 6551 KB)

## References

[1] Y. Zhang, K. Ruan, K. Zhou, J. Gu, Controlled distributed Ti_3_C_2_T_x_ hollow microspheres on thermally conductive polyimide composite films for excellent electromagnetic interference shielding. Adv. Mater. **35**, 2211642 (2023). 10.1002/adma.202211642 10.1002/adma.202211642 36703618

[2] Y. Zhang, J. Gu, A perspective for developing polymer-based electromagnetic interference shielding composites. Nano-Micro Lett. **14**, 89 (2022). 10.1007/s40820-022-00843-3 10.1007/s40820-022-00843-3 PMC8976017 35362900

[3] H. Lv, Z. Yang, H. Pan, R. Wu, Electromagnetic absorption materials: Current progress and new frontiers. Prog. Mater. Sci. **127**, 100946 (2022). 10.1016/j.pmatsci.2022.100946

[4] Z. Wu, H.-W. Cheng, C. Jin, B. Yang, C. Xu et al., Dimensional design and core-shell engineering of nanomaterials for electromagnetic wave absorption. Adv. Mater. **34**, e2107538 (2022). 10.1002/adma.202107538 34755916 10.1002/adma.202107538

[5] M. Sun, D. Wang, Z. Xiong, Z. Zhang, L. Qin et al., Multi-dimensional Ni@C-CoNi composites with strong magnetic interaction toward superior microwave absorption. J. Mater. Sci. Technol. **130**, 176–183 (2022). 10.1016/j.jmst.2022.05.016

[6] Y. Zhang, J. Kong, J. Gu, New generation electromagnetic materials: harvesting instead of dissipation solo. Sci. Bull. **67**, 1413–1415 (2022). 10.1016/j.scib.2022.06.017 10.1016/j.scib.2022.06.017 36546181

[7] Y. Guo, K. Ruan, G. Wang, J. Gu, Advances and mechanisms in polymer composites toward thermal conduction and electromagnetic wave absorption. Sci. Bull. **68**, 1195–1212 (2023). 10.1016/j.scib.2023.04.036 10.1016/j.scib.2023.04.036 37179235

[8] Y. Lian, B. Han, D. Liu, Y. Wang, H. Zhao et al., Solvent-free synthesis of ultrafine tungsten carbide nanoparticles-decorated carbon nanosheets for microwave absorption. Nano-Micro Lett. **12**, 153 (2020). 10.1007/s40820-020-00491-5 10.1007/s40820-020-00491-5 PMC7770940 34138171

[9] X. Wang, F. Pan, Z. Xiang, Q. Zeng, K. Pei et al., Magnetic vortex core-shell Fe_3_O_4_@C nanorings with enhanced microwave absorption performance. Carbon **157**, 130–139 (2020). 10.1016/j.carbon.2019.10.030

[10] Z. Cheng, R. Wang, Y. Cao, Z. Cai, Z. Zhang et al., Intelligent off/on switchable microwave absorption performance of reduced graphene oxide/VO_2_ composite aerogel. Adv. Funct. Mater. **32**, 2205160 (2022). 10.1002/adfm.202205160

[11] H.-Y. Wang, X.-B. Sun, S.-H. Yang, P.-Y. Zhao, X.-J. Zhang et al., 3D ultralight hollow NiCo Compound@MXene composites for tunable and high-efficient microwave absorption. Nano-Micro Lett. **13**, 206 (2021). 10.1007/s40820-021-00727-y 10.1007/s40820-021-00727-y PMC8505608 34633551

[12] P. Miao, N. Qu, W. Chen, T. Wang, W. Zhao et al., A two-dimensional semiconductive Cu-S metal-organic framework for broadband microwave absorption. Chem. Eng. J. **454**, 140445 (2023). 10.1016/j.cej.2022.140445

[13] P. Liu, S. Gao, X. Liu, Y. Huang, W. He et al., Rational construction of hierarchical hollow CuS@CoS_2_ nanoboxes with heterogeneous interfaces for high-efficiency microwave absorption materials. Compos. Part B Eng. **192**, 107992 (2020). 10.1016/j.compositesb.2020.107992

[14] M. Ning, Q. Man, G. Tan, Z. Lei, J. Li et al., Ultrathin MoS_2_ nanosheets encapsulated in hollow carbon spheres: a case of a dielectric absorber with optimized impedance for efficient microwave absorption. ACS Appl. Mater. Interfaces **12**, 20785–20796 (2020). 10.1021/acsami.9b20433 32285661 10.1021/acsami.9b20433

[15] Q. Liu, Q. Cao, H. Bi, C. Liang, K. Yuan et al., CoNi@SiO_2_ @TiO_2_ and CoNi@Air@TiO_2_ microspheres with strong wideband microwave absorption. Adv. Mater. **28**, 486–490 (2016). 10.1002/adma.201503149 26588359 10.1002/adma.201503149

[16] C. Wen, X. Li, R. Zhang, C. Xu, W. You et al., High-density anisotropy magnetism enhanced microwave absorption performance in Ti_3_C_2_T_*x*_ MXene@Ni microspheres. ACS Nano **16**, 1150–1159 (2022). 10.1021/acsnano.1c08957 34957827 10.1021/acsnano.1c08957

[17] B. Zhao, Y. Li, Q. Zeng, L. Wang, J. Ding et al., Galvanic replacement reaction involving core-shell magnetic chains and orientation-tunable microwave absorption properties. Small **16**, e2003502 (2020). 10.1002/smll.202003502 32893495 10.1002/smll.202003502

[18] L. Cui, Y. Wang, X. Han, P. Xu, F. Wang et al., Phenolic resin reinforcement: a new strategy for hollow NiCo@C microboxes against electromagnetic pollution. Carbon **174**, 673–682 (2021). 10.1016/j.carbon.2020.10.070

[19] Q. Liang, L. Wang, X. Qi, Q. Peng, X. Gong et al., Hierarchical engineering of CoNi@Air@C/SiO_2_@Polypyrrole multicomponent nanocubes to improve the dielectric loss capability and magnetic-dielectric synergy. J. Mater. Sci. Technol. **147**, 37–46 (2023). 10.1016/j.jmst.2022.10.069

[20] B. Cai, L. Zhou, P.-Y. Zhao, H.-L. Peng, Z.-L. Hou et al., Interface-induced dual-pinning mechanism enhances low-frequency electromagnetic wave loss. Nat. Commun. **15**, 3299 (2024). 10.1038/s41467-024-47537-5 38632245 10.1038/s41467-024-47537-5 PMC11024160

[21] M. Huang, L. Wang, W. You, R. Che, Single zinc atoms anchored on MOF-derived N-doped carbon shell cooperated with magnetic core as an ultrawideband microwave absorber. Small **17**, e2101416 (2021). 10.1002/smll.202101416 34159720 10.1002/smll.202101416

[22] Z. Li, X. Han, Y. Ma, D. Liu, Y. Wang et al., MOFs-derived hollow Co/C microspheres with enhanced microwave absorption performance. ACS Sustainable Chem. Eng. **6**, 8904–8913 (2018). 10.1021/acssuschemeng.8b01270

[23] P. Liu, S. Gao, Y. Wang, F. Zhou, Y. Huang et al., Metal-organic polymer coordination materials derived Co/N-doped porous carbon composites for frequency-selective microwave absorption. Compos. Part B Eng. **202**, 108406 (2020). 10.1016/j.compositesb.2020.108406

[24] N. Wu, B. Zhao, Y. Lian, S. Liu, Y. Xian et al., Metal organic frameworks derived Ni_x_Se_y_@NC hollow microspheres with modifiable composition and broadband microwave attenuation. Carbon **226**, 119215 (2024). 10.1016/j.carbon.2024.119215

[25] R. Qiang, Y. Du, H. Zhao, Y. Wang, C. Tian et al., Metal organic framework-derived Fe/C nanocubes toward efficient microwave absorption. J. Mater. Chem. A **3**, 13426–13434 (2015). 10.1039/C5TA01457C

[26] C. Wei, L. Shi, M. Li, M. He, M. Li et al., Hollow engineering of sandwich NC@Co/NC@MnO_2_ composites toward strong wideband electromagnetic wave attenuation. J. Mater. Sci. Technol. **175**, 194–203 (2024). 10.1016/j.jmst.2023.08.020

[27] L. Wang, X. Li, X. Shi, M. Huang, X. Li et al., Recent progress of microwave absorption microspheres by magnetic-dielectric synergy. Nanoscale **13**, 2136–2156 (2021). 10.1039/d0nr06267g 33471004 10.1039/d0nr06267g

[28] Z. He, H. Xu, L. Shi, X. Ren, J. Kong et al., Hierarchical Co_2_ P/CoS_2_@C@MoS_2_ composites with hollow cavity and multiple phases toward wideband electromagnetic wave absorption. Small **20**, e2306253 (2024). 10.1002/smll.202306253 37771205 10.1002/smll.202306253

[29] P. Liu, S. Zheng, Z. He, C. Qu, L. Zhang et al., Optimizing integrated-loss capacities via asymmetric electronic environments for highly efficient electromagnetic wave absorption. Small (2024). 10.1002/smll.202403903 10.1002/smll.202403903 38953301

[30] P. Liu, S. Gao, G. Zhang, Y. Huang, W. You et al., Hollow engineering to Co@N-doped carbon nanocages via synergistic protecting-etching strategy for ultrahigh microwave absorption. Adv. Funct. Mater. **31**, 2102812 (2021). 10.1002/adfm.202102812

[31] W. Wang, K. Nan, H. Zheng, Q. Li, Y. Wang, Ion-exchange reaction construction of carbon nanotube-modified CoNi@MoO_2_/C composite for ultra-intense and broad electromagnetic wave absorption. Carbon **210**, 118074 (2023). 10.1016/j.carbon.2023.118074

[32] J. Gao, Y. Hu, Y. Wang, X. Lin, K. Hu et al., MOF structure engineering to synthesize Co-N-C catalyst with richer accessible active sites for enhanced oxygen reduction. Small **17**, e2104684 (2021). 10.1002/smll.202104684 34738730 10.1002/smll.202104684

[33] Z.-L. Hou, X. Gao, J. Zhang, G. Wang, A perspective on impedance matching and resonance absorption mechanism for electromagnetic wave absorbing. Carbon **222**, 118935 (2024). 10.1016/j.carbon.2024.118935

[34] S. Kang, W. Zhang, Z. Hu, J. Yu, Y. Wang et al., Porous core-shell zeolitic imidazolate framework-derived Co/NPC@ZnO-decorated reduced graphene oxide for lightweight and broadband electromagnetic wave absorber. J. Alloys Compd. **818**, 152932 (2020). 10.1016/j.jallcom.2019.152932

[35] Y. Wang, X. Di, X. Wu, X. Li, MOF-derived nanoporous carbon/Co/Co_3_O_4_/CNTs/RGO composite with hierarchical structure as a high-efficiency electromagnetic wave absorber. J. Alloys Compd. **846**, 156215 (2020). 10.1016/j.jallcom.2020.156215

[36] L. Gai, Y. Wang, P. Wan, S. Yu, Y. Chen et al., Compositional and hollow engineering of silicon carbide/carbon microspheres as high-performance microwave absorbing materials with good environmental tolerance. Nano-Micro Lett. **16**, 167 (2024). 10.1007/s40820-024-01369-6 10.1007/s40820-024-01369-6 PMC10987424 38564086

[37] Y. Han, M. He, J. Hu, P. Liu, Z. Liu et al., Hierarchical design of FeCo-based microchains for enhanced microwave absorption in C band. Nano Res. **16**, 1773–1778 (2023). 10.1007/s12274-022-5111-y

[38] Y. Huang, S.L. Zhang, X.F. Lu, Z.P. Wu, D. Luan et al., Trimetallic spinel NiCo_2-__*x*_Fe_*x*_O_4_ nanoboxes for highly efficient electrocatalytic oxygen evolution. Angew. Chem. Int. Ed. Engl. **60**, 11841–11846 (2021). 10.1002/anie.202103058 33739587 10.1002/anie.202103058

[39] Y.-L. Wang, P.-Y. Zhao, B.-L. Liang, K. Chen, G.-S. Wang, Carbon nanotubes decorated Co/C from ZIF-67/melamine as high efficient microwave absorbing material. Carbon **202**, 66–75 (2023). 10.1016/j.carbon.2022.10.043

[40] C. Lei, J. Li, Y. Wu, Y. Xie, Y. Ling et al., Construction of gradient hierarchical and hetero-interfaces structure for ultra-broad microwave absorption. Nano Mater. Sci. (2024). 10.1016/j.nanoms.2024.04.004

[41] I. Lorite, J.J. Romero, J.F. Fernández, Effects of the agglomeration state on the Raman properties of Co_3_O_4_ nanoparticles. J. Raman Spectrosc. **43**, 1443–1448 (2012). 10.1002/jrs.4098

[42] M. Qin, L. Zhang, X. Zhao, H. Wu, Defect induced polarization loss in multi-shelled spinel hollow spheres for electromagnetic wave absorption application. Adv. Sci. **8**, 2004640 (2021). 10.1002/advs.202004640 10.1002/advs.202004640 PMC8061380 33898201

[43] J.-C. Shu, X.-Y. Huang, M.-S. Cao, Assembling 3D flower-like Co_3_O_4_-MWCNT architecture for optimizing low-frequency microwave absorption. Carbon **174**, 638–646 (2021). 10.1016/j.carbon.2020.11.087

[44] X. Xie, C. Ni, Z. Lin, D. Wu, X. Sun et al., Phase and morphology evolution of high dielectric CoO/Co_3_O_4_ particles with Co_3_O_4_ nanoneedles on surface for excellent microwave absorption application. Chem. Eng. J. **396**, 125205 (2020). 10.1016/j.cej.2020.125205

[45] J. Cheng, H. Jiang, L. Cai, F. Pan, Y. Shi et al., Porous N-doped C/VB-group VS_2_ composites derived from perishable garbage to synergistically solve the environmental and electromagnetic pollution. Chem. Eng. J. **457**, 141208 (2023). 10.1016/j.cej.2022.141208

[46] Y. Cheng, J.Z.Y. Seow, H. Zhao, Z.J. Xu, G. Ji, A flexible and lightweight biomass-reinforced microwave absorber. Nano-Micro Lett. **12**, 125 (2020). 10.1007/s40820-020-00461-x 10.1007/s40820-020-00461-x PMC7770825 34138152

[47] G. Liu, J. Tu, C. Wu, Y. Fu, C. Chu et al., High-yield two-dimensional metal-organic framework derivatives for wideband electromagnetic wave absorption. ACS Appl. Mater. Interfaces **13**, 20459–20466 (2021). 10.1021/acsami.1c00281 33890473 10.1021/acsami.1c00281

[48] J. Chen, J. Zheng, F. Wang, Q. Huang, G. Ji, Carbon fibers embedded with FeIII-MOF-5-derived composites for enhanced microwave absorption. Carbon **174**, 509–517 (2021). 10.1016/j.carbon.2020.12.077

[49] W. Wang, K. Nan, H. Zheng, Q. Li, Y. Wang, Heterostructure design of hydrangea-like Co_2_P/Ni_2_P@C multilayered hollow microspheres for high-efficiency microwave absorption. J. Mater. Sci. Technol. **181**, 104–114 (2024). 10.1016/j.jmst.2023.09.023

[50] J. Chen, J. Zheng, Q. Huang, F. Wang, G. Ji, Enhanced microwave absorbing ability of carbon fibers with embedded FeCo/CoFe_2_O_4_ nanoparticles. ACS Appl. Mater. Interfaces **13**, 36182–36189 (2021). 10.1021/acsami.1c09430 34291899 10.1021/acsami.1c09430

[51] X. Zhong, M. He, C. Zhang, Y. Guo, J. Hu et al., Heterostructured BN@Co-C@C endowing polyester composites excellent thermal conductivity and microwave absorption at C band. Adv. Funct. Mater. **34**, 2313544 (2024). 10.1002/adfm.202313544

[52] B. Zhao, Y. Li, Q. Zeng, B. Fan, L. Wang et al., Growth of magnetic metals on carbon microspheres with synergetic dissipation abilities to broaden microwave absorption. J. Mater. Sci. Technol. **107**, 100–110 (2022). 10.1016/j.jmst.2021.07.044

[53] P. Liu, Y. Li, H. Xu, L. Shi, J. Kong et al., Hierarchical Fe-Co@TiO_2_ with incoherent heterointerfaces and gradient magnetic domains for electromagnetic wave absorption. ACS Nano **18**, 560–570 (2024). 10.1021/acsnano.3c08569 38109426 10.1021/acsnano.3c08569

[54] M. He, J. Hu, H. Yan, X. Zhong, Y. Zhang et al., Shape anisotropic chain-like CoNi/polydimethylsiloxane composite films with excellent low-frequency microwave absorption and high thermal conductivity. Adv. Funct. Mater. (2024). 10.1002/adfm.202316691 10.1002/adma.202410186 39380425

[55] B. Quan, W. Gu, J. Sheng, X. Lv, Y. Mao et al., From intrinsic dielectric loss to geometry patterns: Dual-principles strategy for ultrabroad band microwave absorption. Nano Res. **14**, 1495–1501 (2021). 10.1007/s12274-020-3208-8

