# Supplementary material for: Low-Temperature Oxidation Induced Phase Evolution with Gradient Magnetic Heterointerfaces for Superior Electromagnetic Wave Absorption
Source: Nanomicro Lett. 2024 Sep 22;17:7. doi: 10.1007/s40820-024-01516-z (PMC11416442; doi:10.1007/s40820-024-01516-z)
Supplement: Supplementary file 1 — Supplementary file1 (DOCX 6551 KB) [file 40820_2024_1516_MOESM1_ESM.docx]

Supporting Information for

**Low-Temperature Oxidation Induced Phase Evolution with Gradient Magnetic Heterointerfaces for Superior Electromagnetic Wave Absorption**

Zizhuang He^1^, Lingzi Shi^1^, Ran Sun^1,^*, Lianfei Ding^1^, Mukun He^1^, Jiaming Li^1^, Hua Guo^1^, Tiande Gao^2,^*, Panbo Liu^1,^*

^1^School of Chemistry and Chemical Engineering, Northwestern Polytechnical University, Xi’an 710129, P. R. China

^2^School of Marine Science and Technology, Northwestern Polytechnical University, Xi’an 710072, P. R. China

*Corresponding authors. E-mail: [sunran@nwpu.edu.cn](mailto:sunran@nwpu.edu.cn) (Ran Sun), [gaotiande@nwpu.edu.cn](mailto:gaotiande@nwpu.edu.cn) (Tiande Gao), [liupanbo@nwpu.edu.cn](mailto:liupanbo@nwpu.edu.cn) (Panbo Liu)

**S1 Experimental Sections**

**S1.1 Microwave Absorption Measurement**

The samples were dispersed in paraffin matrix with 25 wt%, which were made into a circular ring with an internal diameter of 3.0 mm and an external diameter of 7.0 mm. The *R_L_* values were calculated based on the transmission line theory with the electromagnetic parameters (complex permittivity and complex permeability) measured by an HP8510C vector network analyzer in 2-18 GHz:

| $RL\left( \text{dB} \right)=20\log\left\vert\frac{Z_{in}-Z_{0}}{Z_{in}+Z_{0}} \right\vert$ | (S1) |
| --- | --- |
| $\text{Z}_{\text{in}}=Z_{0}\sqrt{\frac{\mu_{r}}{\varepsilon_{r}}}\tanh\left[ j\left( \frac{2\text{π}fd}{c} \right)\sqrt{\varepsilon_{r}\mu_{r}} \right]$ | (S2) |

Where $Z_{in}$ is the input impedance of the microwave absorbers, $Z_{0}$ is the impedance of free space, *d* is the thickness of the absorbers, $c$ is the speed of light in free space, $f$ is the incident frequency, $\varepsilon_{r}$ and $\mu_{r}$ are the relative complex permittivity and permeability, respectively. $\varepsilon_{r}=\varepsilon'-j\varepsilon''$, $\mu_{r}=\mu'-j\mu''$.

In addition, the attenuation constant *α* is another vital factor for EM absorption, determining the attenuation ability of the absorbers to incident EM waves, which can be escribed as following equations:

| $\alpha=\frac{\sqrt{2}\pi f}{c}\left[ \left( \mu^{''}\varepsilon^{''}-\mu'\varepsilon' \right)+\sqrt{\left( \mu^{''}\varepsilon^{''}-\mu'\varepsilon' \right)^{2}+\left( \mu^{'}\varepsilon^{''}+\mu''\varepsilon' \right)^{2}} \right]^{\frac{1}{2}}$ | (S3) |
| --- | --- |

The magnetic material can respond to the externally altered magnetic field and form induced eddy current. The corresponding loss was termed as eddy current. Eddy current loss involves an energy conversion from magnetic field to electrical energy. In general, the eddy current loss (termed as $C_{0}$) is extremely related to the diameter ($d$) and the conductivity ($\sigma$), which can be approximately expressed as follows:

| $C_{0}=\frac{2\pi\mu_{0}\sigma d^{2}}{3}=\mu''\left( \mu' \right)^{-2}f^{-1}$ | (S4) |
| --- | --- |

**S1.2 Impedance Matching**

The impedance match degree ($Z_{in}/Z_{0}$) is calculated by a above-mentioned method as follows:

| $\frac{Z_{in}}{Z_{0}}=\sqrt{\frac{\mu_{r}}{\varepsilon_{r}}}\tanh\left[ j\left( \frac{2\text{π}fd}{c} \right)\sqrt{\varepsilon_{r}\mu_{r}} \right]$ | (S5) |
| --- | --- |

When $({Z_{in}}/{Z_{0}})$ is between 0.8 and 1.2, it indicates impedance matching.

**S1.3 Debye Relaxation**

According to the Debye theory, the relative complex permittivity can be expressed as follows:

| $\varepsilon_{r}=\varepsilon^{'}-j\varepsilon^{''}=\varepsilon_{\infty}+\frac{\varepsilon_{S}-\varepsilon_{\infty}}{1+j\omega\tau}$ | (S6) |
| --- | --- |

where $\varepsilon_{S}$ is the static dielectric constant, $\varepsilon_{\infty}$ is the dielectric constant at infinite frequency, $\omega=2\pi f$ is the angular frequency, and $\tau$ refer to the polarization relaxation time. In consequence, the $\varepsilon^{'}$ and $\varepsilon^{'}'$ can be described as follows:

| $\varepsilon^{'}=\varepsilon_{\infty}+\frac{\varepsilon_{S}-\varepsilon_{\infty}}{1+\omega^{2}\tau^{2}}$ | (S7) |
| --- | --- |
| $\varepsilon^{''}=\frac{\varepsilon_{S}-\varepsilon_{\infty}}{1+\omega^{2}\tau^{2}}\omega\tau+\frac{\sigma}{\omega\varepsilon_{0}}=\varepsilon_{p}^{''}+\varepsilon_{c}^{''}$ | (S8) |

where $\varepsilon_{p}^{''}$ is polarization loss, $\varepsilon_{c}^{''}$ is conductive loss, $\sigma$ is the conductivity of the material.

**S1.4 Radar Cross Section simulation and calculation**

The RCS calculation equation can be written in the form:

| $\sigma\left( dB\cdot m^{2} \right)=10 log(\frac{4\pi S}{\lambda^{2}}{\vert\frac{E_{s}}{E_{i}}\vert}^{2})$ | (S9) |
| --- | --- |

where$S$, $\lambda$, $E_{s}$and $E_{i}$ represent the area of the object simulation model, the wavelength of the electromagnetic wave, the electric field strength of the scattered wave and the incident wave, respectively.

During the simulation process, aluminum (Al) plate is applied as a PEC layer. As prepared sample is mixed with paraffin and applied as an absorbent coating on the surface of the PEC layer, and the calculations are started when a suitable excitation boundary is given for the composite.

**S2 Results and Discussion**


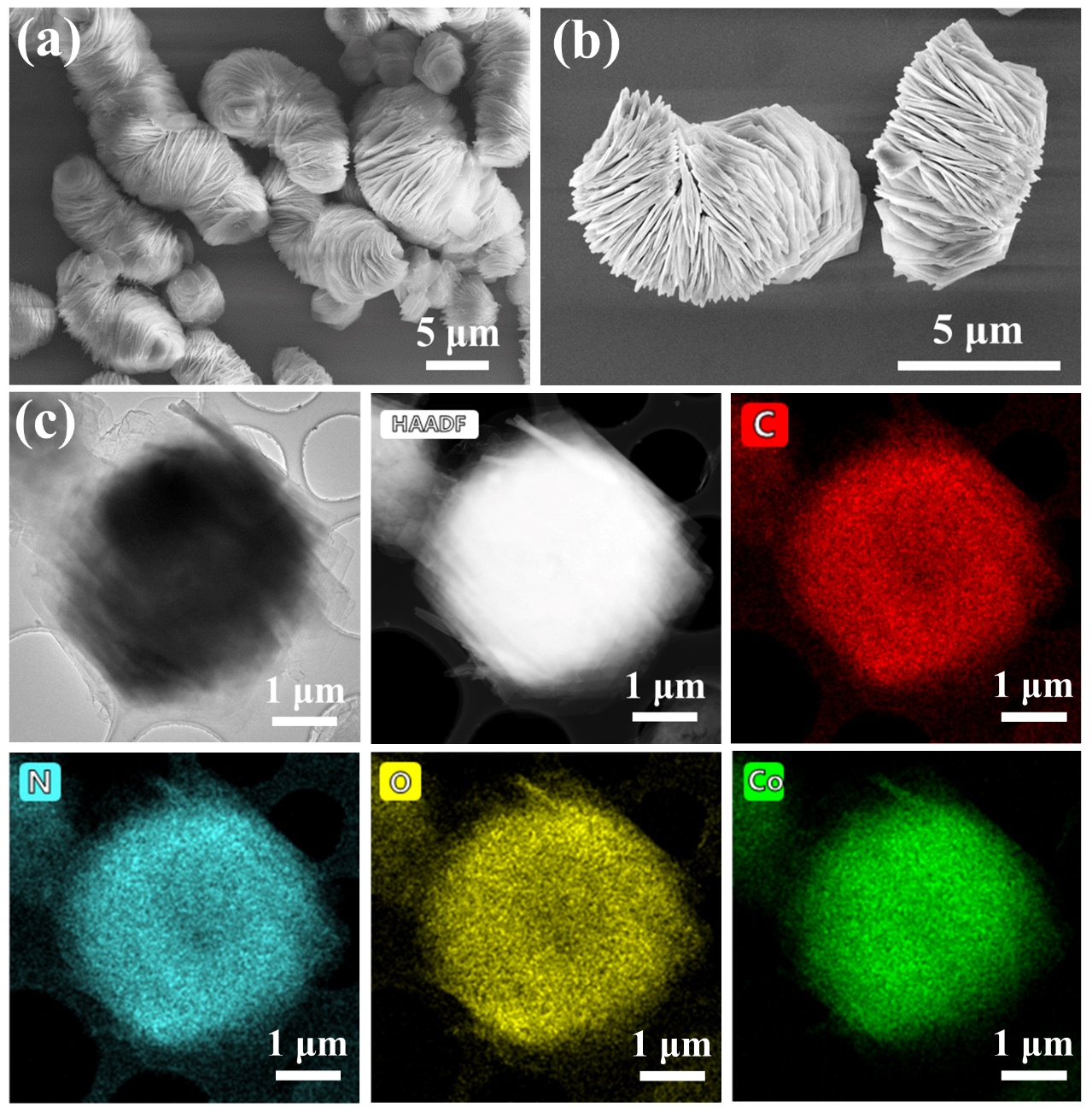


**Fig. S1** TEM image and elemental mapping images of ZIF-accordion


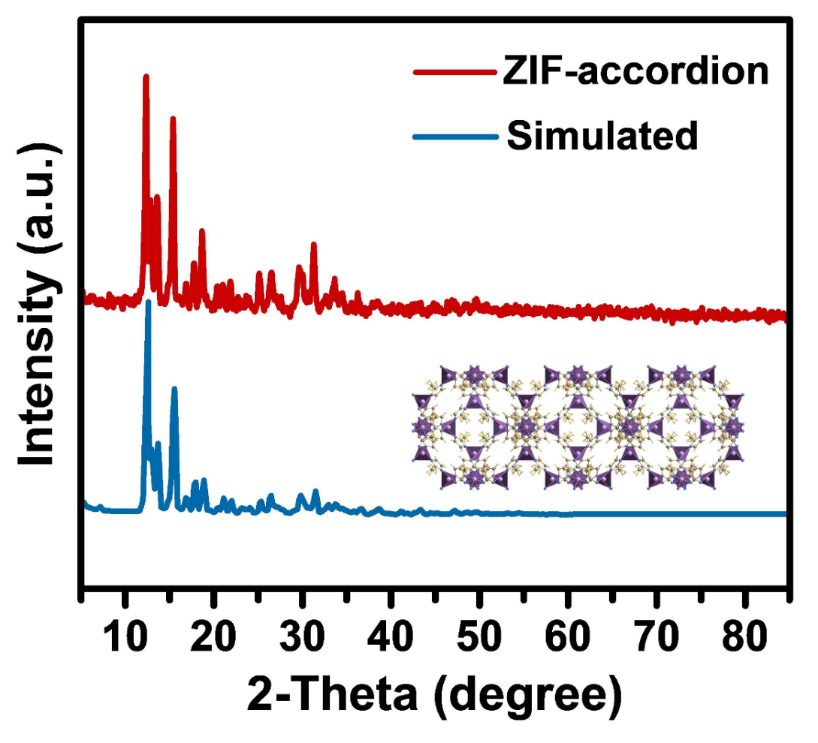


**Fig. S2** XRD pattern of ZIF-accordion (original and simulated)


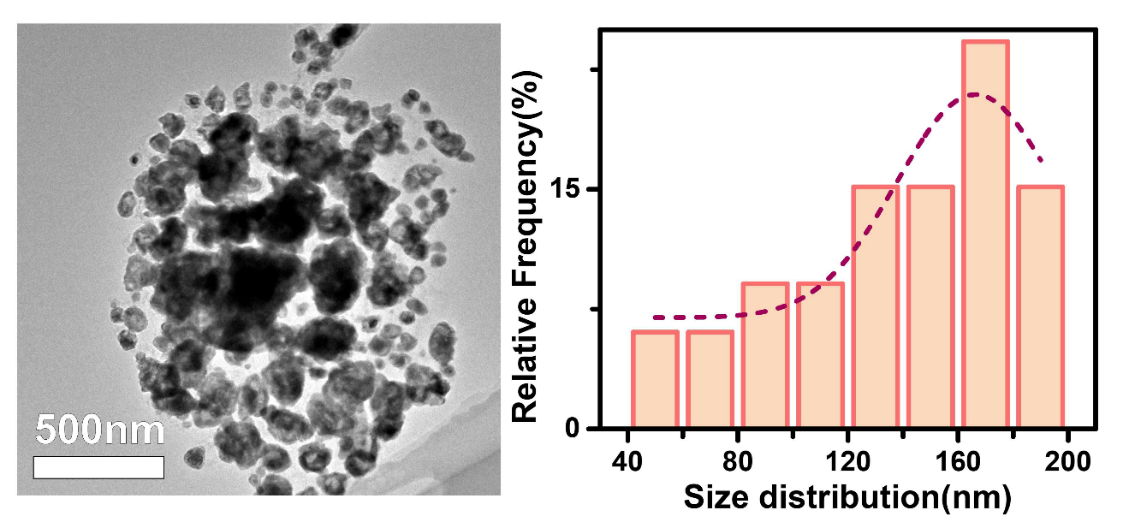


**Fig. S3** TEM image of Co@NC and the size distribution of Co nanoparticles


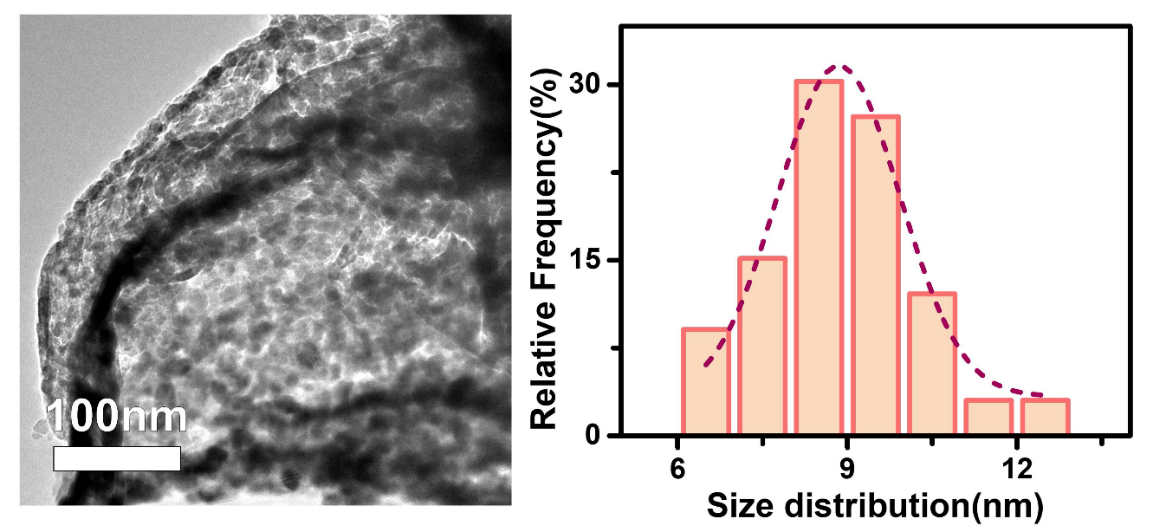


**Fig. S4** TEM image of Co_3_O_4_@NC and the size distribution of Co_3_O_4_ nanoparticles


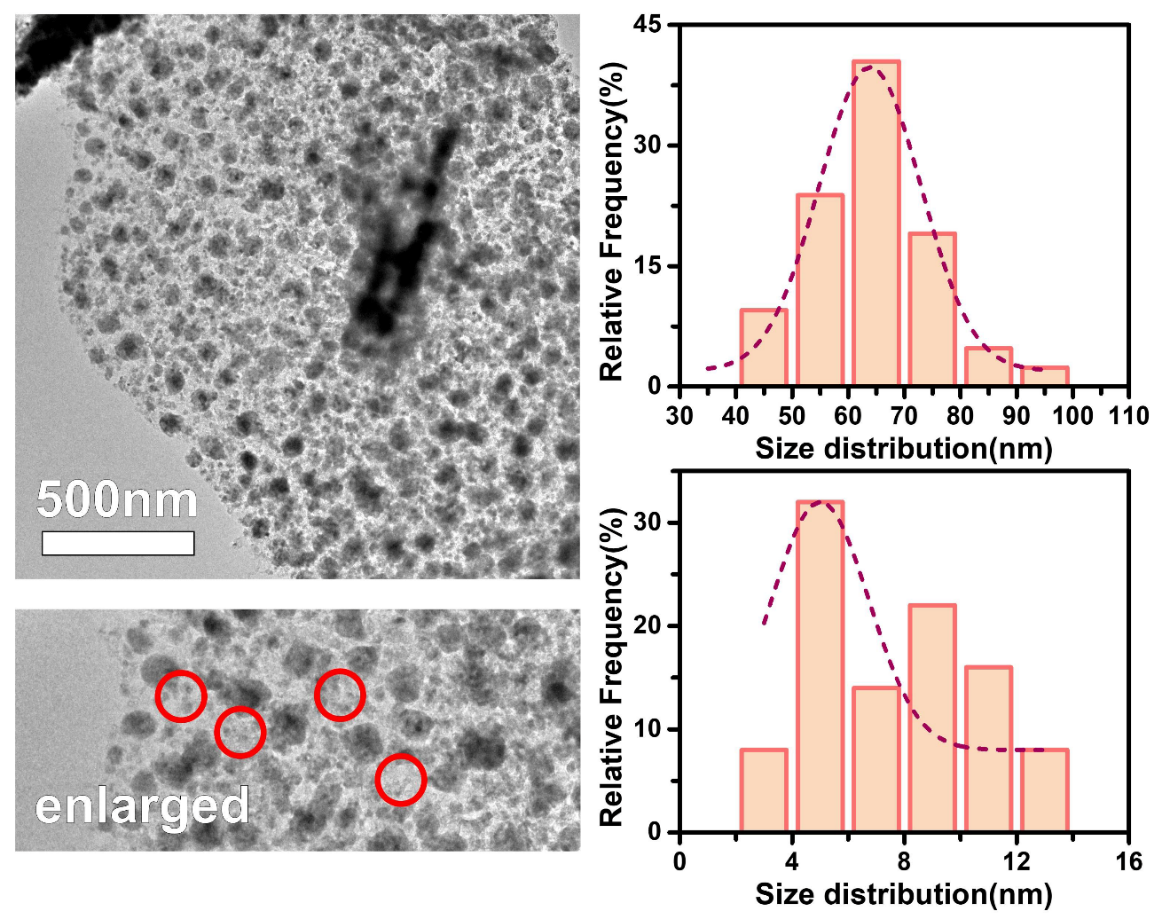


**Fig. S5** TEM image of Co/Co_3_O_4_@NC and the size distribution of Co and Co_3_O_4_ nanoparticles


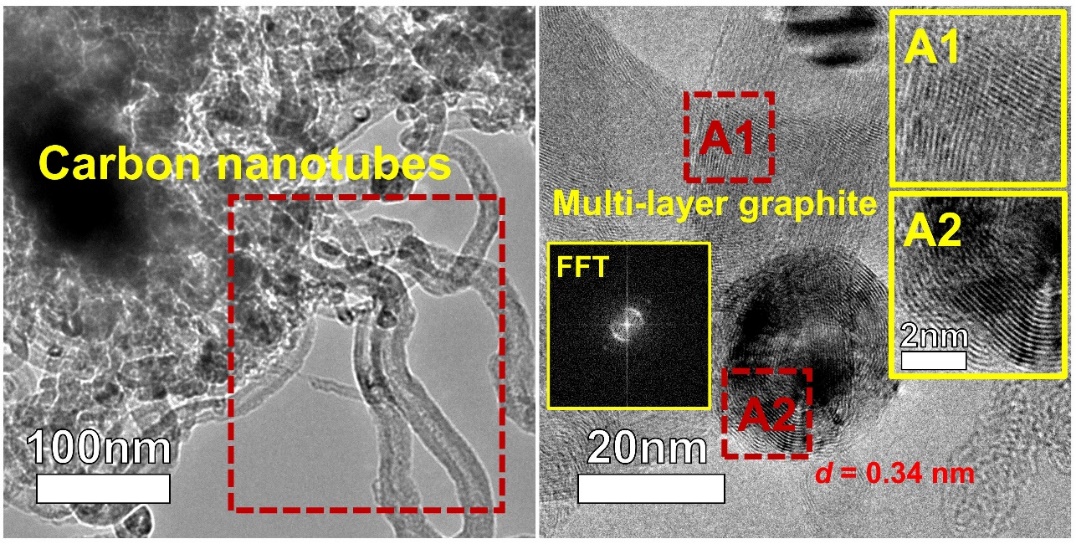


**Fig. S6** TEM image and HRTEM image of carbon nanotubes produced during pyrolysis process


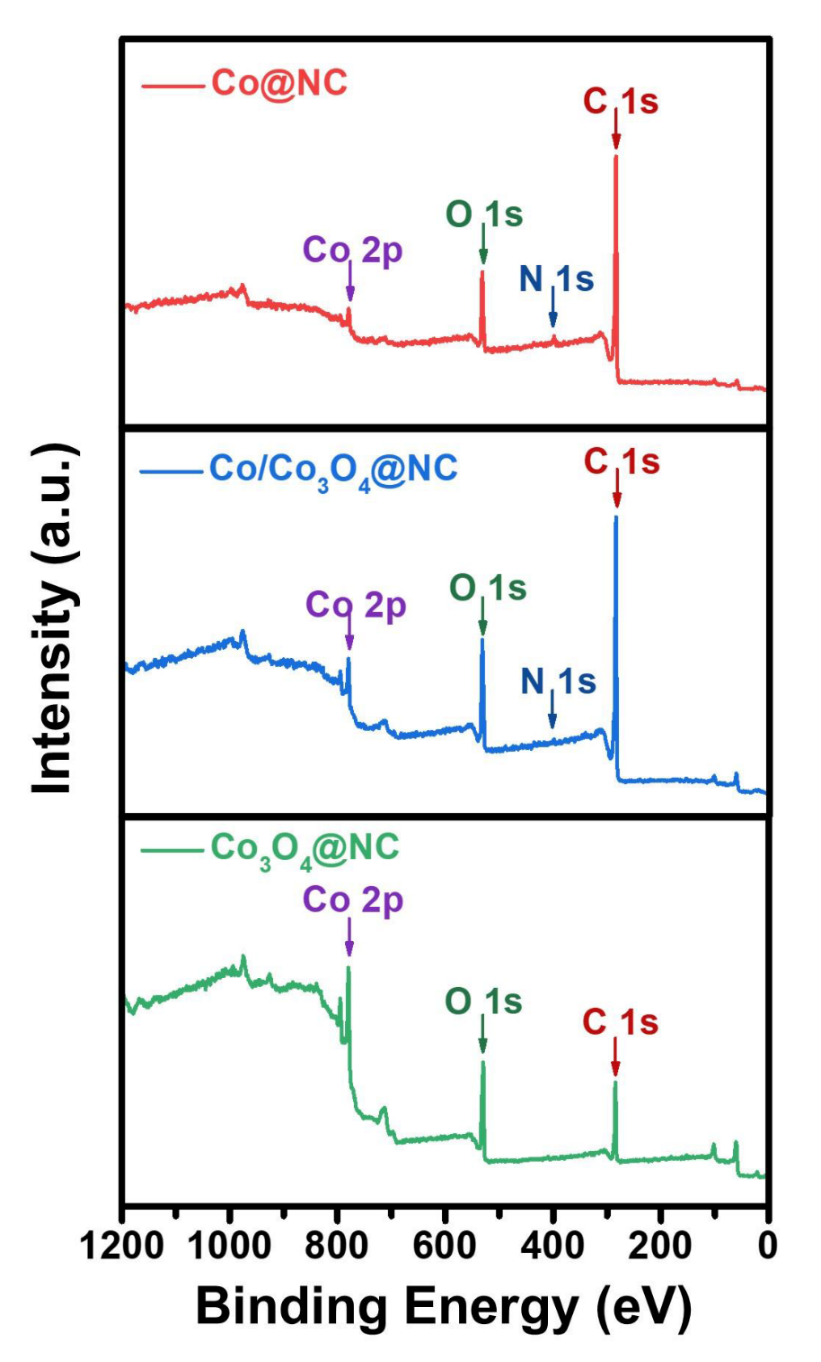


**Fig. S7** XPS spectrum of Co@NC, Co/Co_3_O_4_@NC, Co_3_O_4_@NC


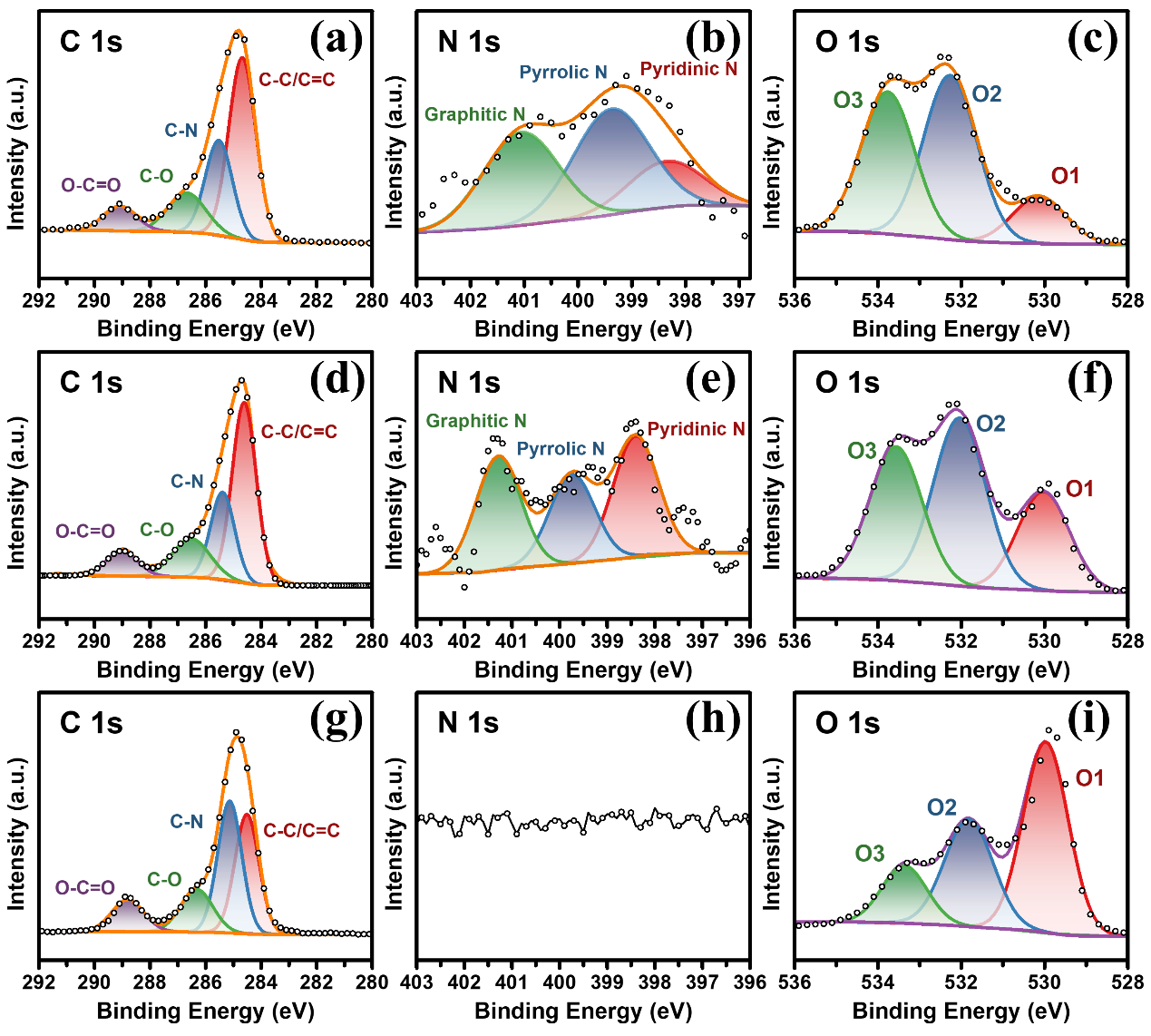


**Fig. S8** C 1s, N 1s and O 1s of **a-c** Co@NC, **d-f** Co/Co_3_O_4_@NC, **g-i** Co_3_O_4_@NC


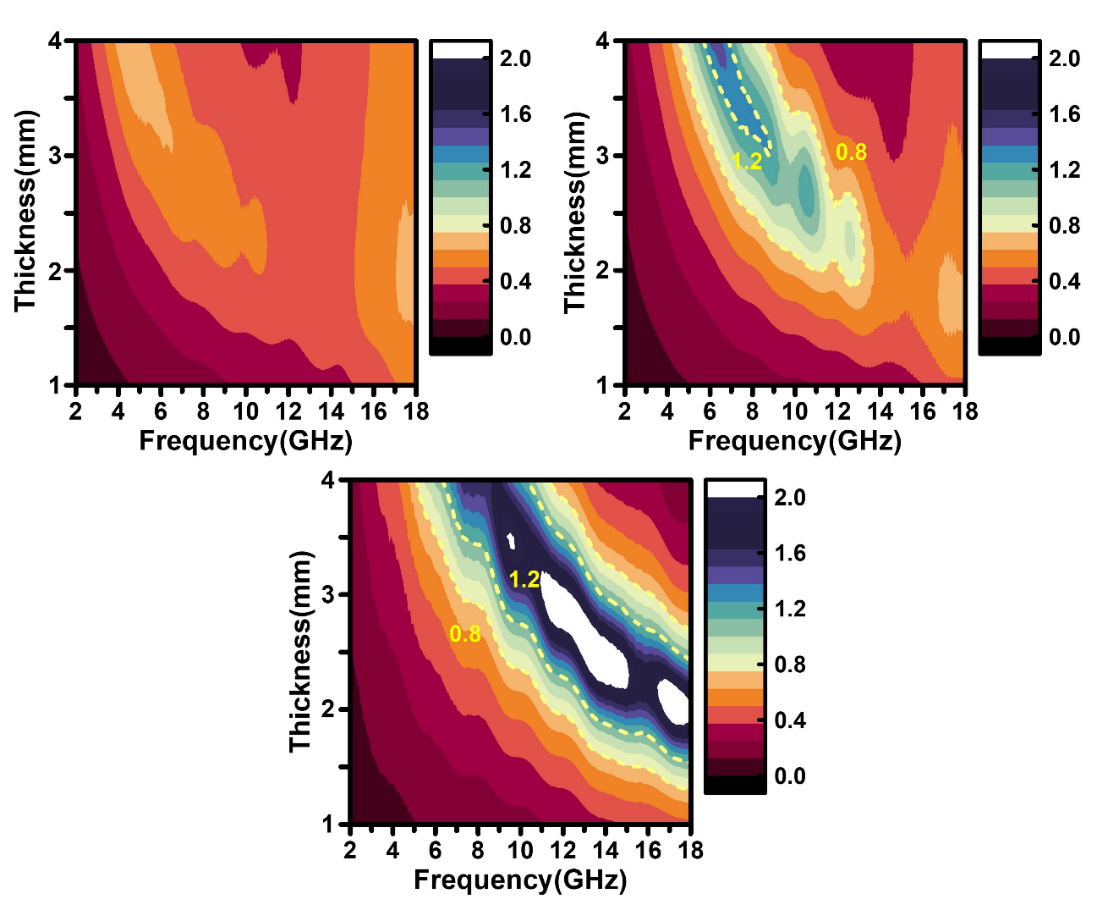


**Fig. S9** 2D impedance matching diagrams of Co@NC, Co/Co_3_O_4_@NC, Co_3_O_4_@NC


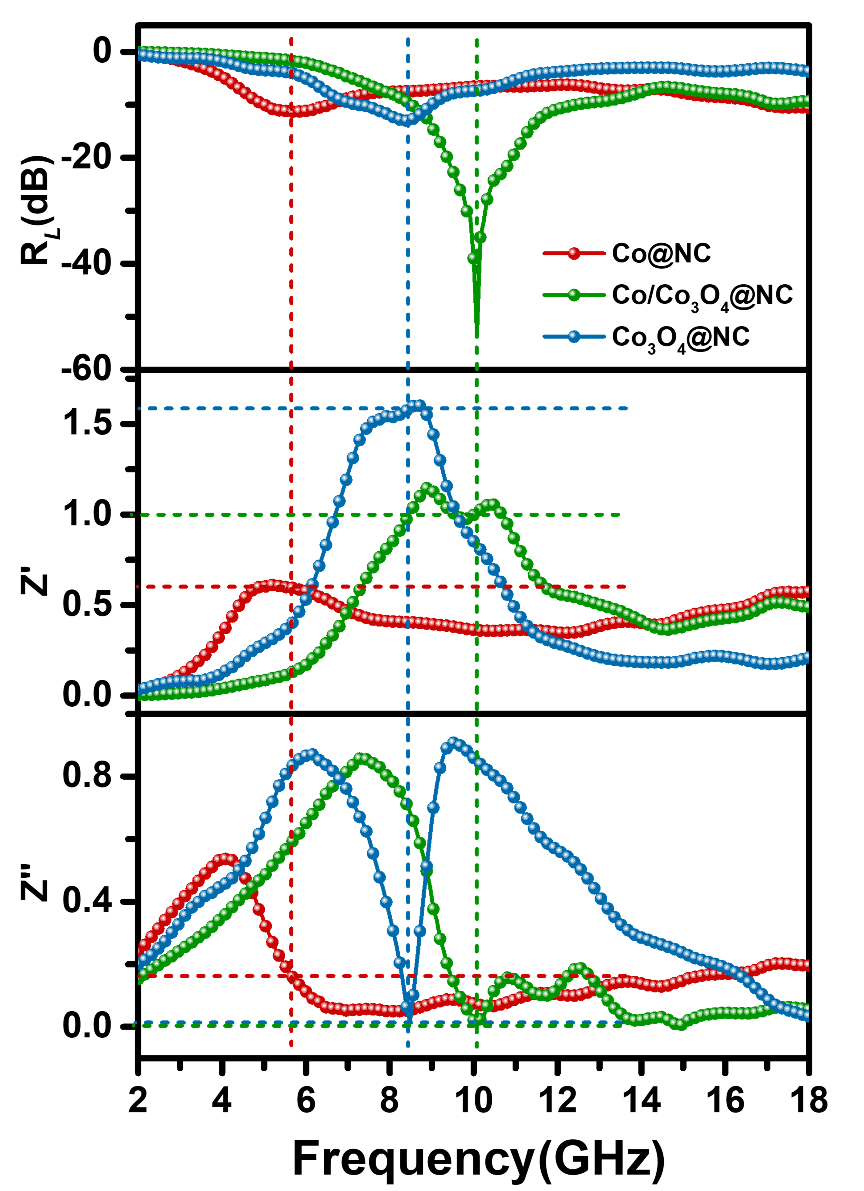


**Fig. S10**  Impedance matching diagrams of Co@NC, Co/Co_3_O_4_@NC, Co_3_O_4_@NC


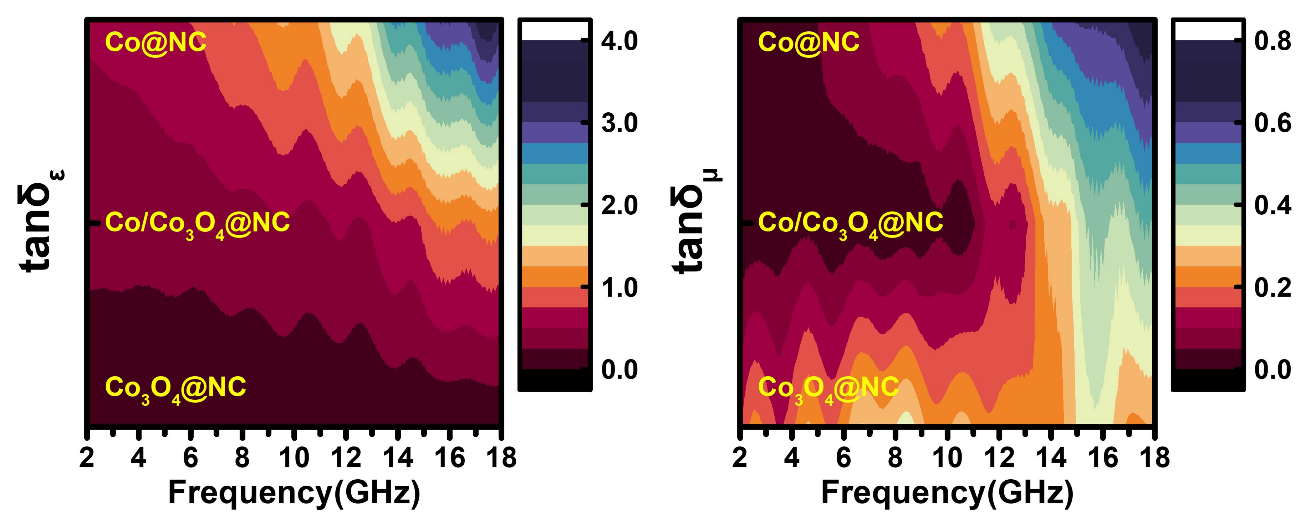


**Fig. S11** The dielectric loss tangent and magnetic loss tangent values of Co@NC, Co/Co_3_O_4_@NC, Co_3_O_4_@NC


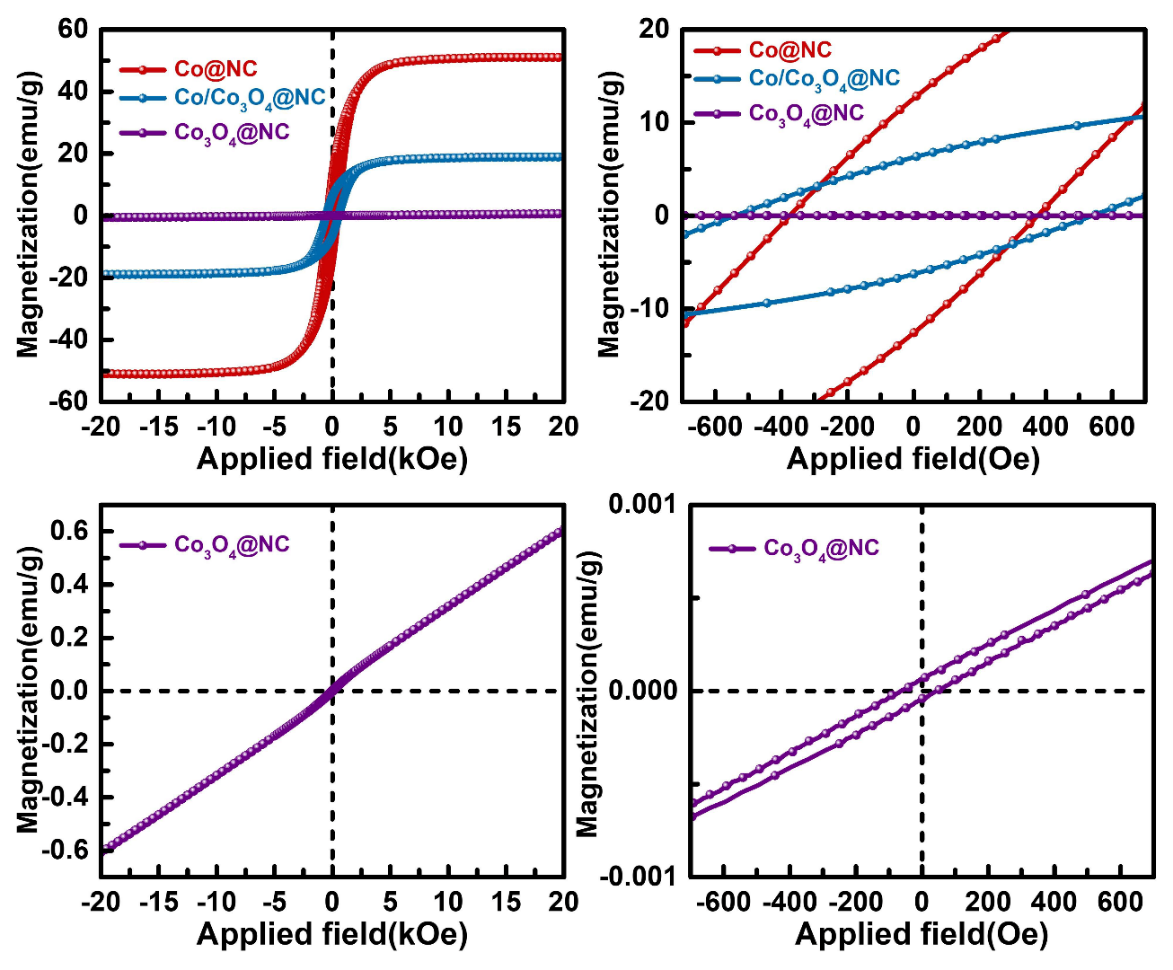


**Fig. S12** Hysteresis loops diagram of Co@NC, Co/Co_3_O_4_@NC, Co_3_O_4_@NC


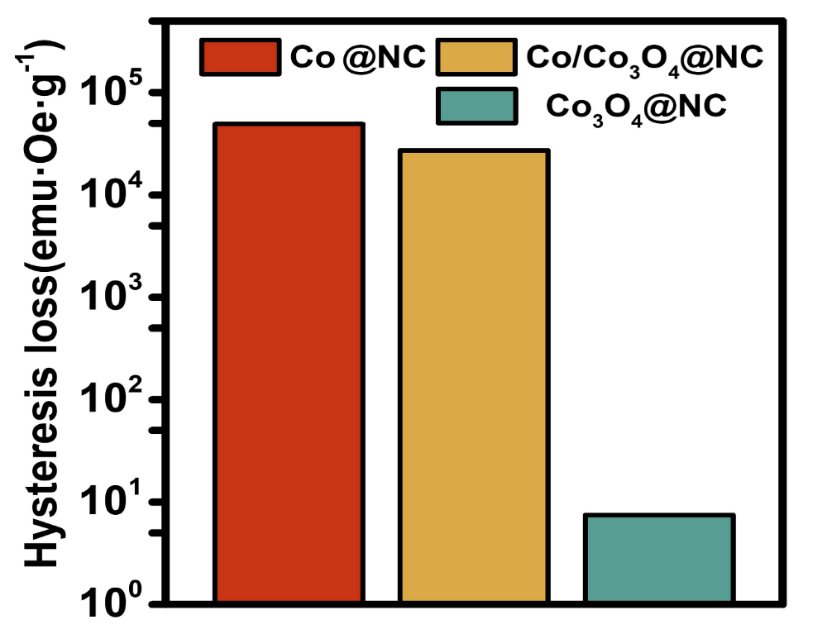


**Fig. S13** The hysteresis loops area integral values of Co@NC, Co/Co_3_O_4_@NC, Co_3_O_4_@NC


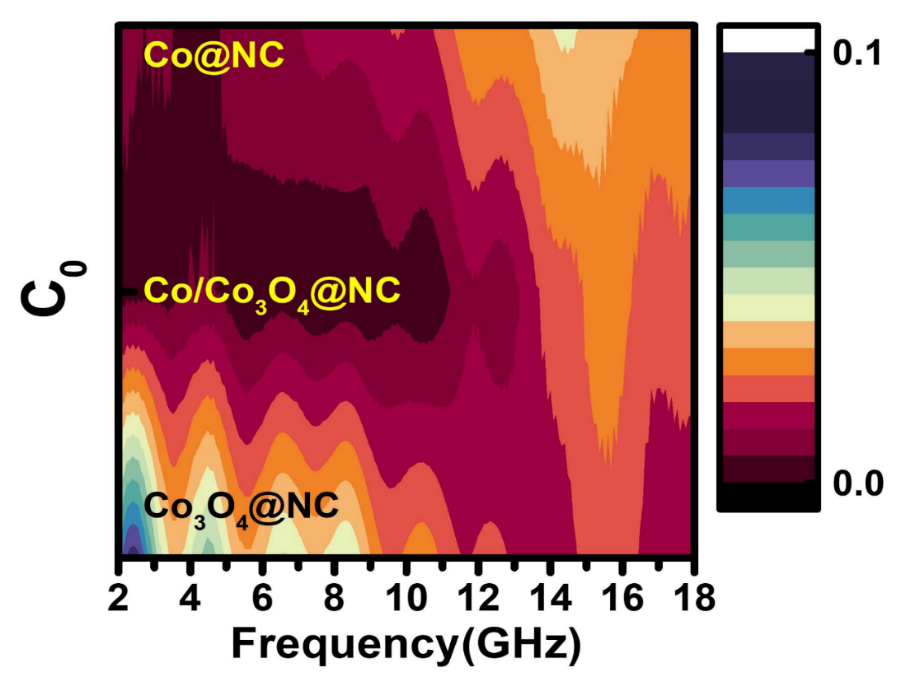


**Fig. S14** μ''(μ')^−2^f^−1^ values of Co@NC, Co/Co_3_O_4_@NC, Co_3_O_4_@NC

**
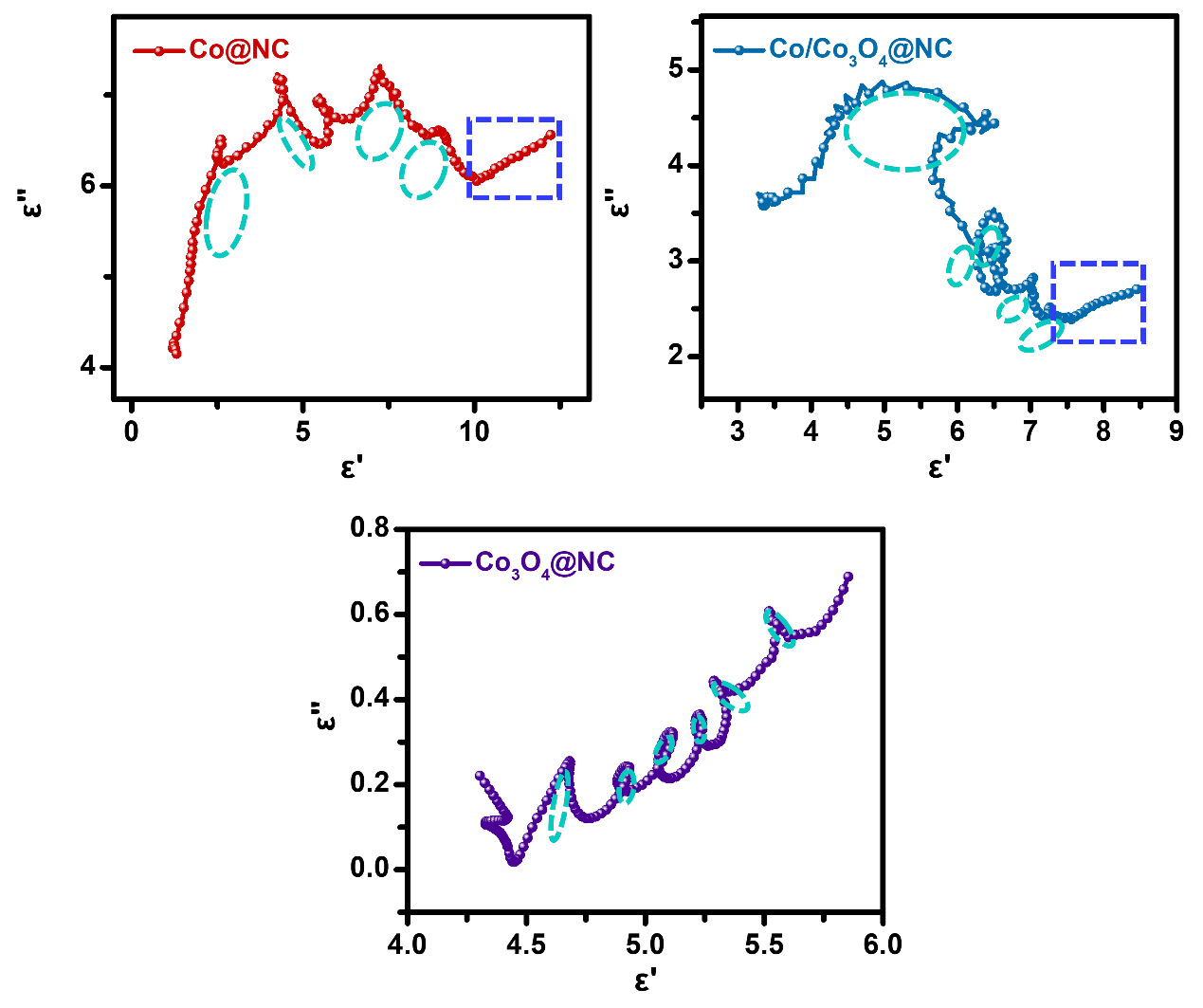
**

**Fig. S15** Cole-Cole curves of Co@NC, Co/Co_3_O_4_@NC, Co_3_O_4_@NC
